# A Computer-Aided Approach for the Discovery of D-Peptides as Inhibitors of SARS-CoV-2 Main Protease

**DOI:** 10.3389/fmolb.2021.816166

**Published:** 2022-01-24

**Authors:** Jorge E. Hernández González, Raphael J. Eberle, Dieter Willbold, Mônika A. Coronado

**Affiliations:** ^1^ Multiuser Center for Biomolecular Innovation, IBILCE, Universidade Estadual Paulista (UNESP), São Jose do Rio Preto, Brazil; ^2^ Laboratory for Molecular Modeling and Dynamics, Instituto de Biofísica Carlos Chagas Filho, Universidade Federal do Rio de Janeiro, Cidade Universitária Ilha do Fundão, Rio de Janeiro, Brazil; ^3^ Institute of Biological Information Processing (IBI-7, Structural Biochemistry), Forschungszentrum Jülich, Jülich, Germany; ^4^ Institut für Physikalische Biologie, Heinrich-Heine-Universität Düsseldorf, Universitätsstraße, Düsseldorf, Germany; ^5^ JuStruct: Jülich Centre for Structural Biology, Forschungszentrum Jülich, Jülich, Germany

**Keywords:** SARS-CoV-2, 3CL^pro^, D-peptide, virtual screening, molecular dynamics simulation

## Abstract

The SARS-CoV-2 main protease, also known as 3-chymotrypsin-like protease (3CL^pro^), is a cysteine protease responsible for the cleavage of viral polyproteins pp1a and pp1ab, at least, at eleven conserved sites, which leads to the formation of mature nonstructural proteins essential for the replication of the virus. Due to its essential role, numerous studies have been conducted so far, which have confirmed 3CL^pro^ as an attractive drug target to combat Covid-19 and have reported a vast number of inhibitors and their co-crystal structures. Despite all the ongoing efforts, D-peptides, which possess key advantages over L-peptides as therapeutic agents, have not been explored as potential drug candidates against 3CL^pro^. The current work fills this gap by reporting an *in silico* approach for the discovery of D-peptides capable of inhibiting 3CL^pro^ that involves structure-based virtual screening (SBVS) of an *in-house* library of D-tripeptides and D-tetrapeptides into the protease active site and subsequent rescoring steps, including Molecular Mechanics Generalized-Born Surface Area (MM-GBSA) free energy calculations and molecular dynamics (MD) simulations. *In vitro* enzymatic assays conducted for the four top-scoring D-tetrapeptides at 20 μM showed that all of them caused 55–85% inhibition of 3CL^pro^ activity, thus highlighting the suitability of the devised approach. Overall, our results present a promising computational strategy to identify D-peptides capable of inhibiting 3CL^pro^, with broader application in problems involving protein inhibition.

## Introduction

Covid-19 is a pandemic disease caused by the novel acute respiratory syndrome coronavirus 2 (SARS-CoV-2). As of December 12th, 2021, over 269 million confirmed Covid-19 cases and 5.3 million related deaths had been reported since the start of the pandemic (World Health Organization, 2021). SARS-CoV-2, together with SARS-CoV and Middle East Respiratory Syndrome (MERS) coronaviruses responsible for two significant outbreaks during the current century, are enveloped and single-stranded RNA viruses (Payne, 2017; Wu F. et al., 2020; Wang et al., 2020). During their replication, coronaviruses encode several accessory proteins and two replicase polyproteins (pp1a and pp1ab) (Marra et al., 2003; Rota et al., 2003; Ziebuhr, 2005; Wu F. et al., 2020; Yan and Wu, 2021), which are proteolytically processed by two cysteine proteases, i.e., the papain-like protease (PL^pro^) and the main protease, also called 3-chymotrypsin-like protease (3CL^pro^). The latter cleaves the pp1a and pp1b at 11 conserved sites by recognizing the XXXLQAXXX and XXXLQSXXX sequence motifs, thus generating nonstructural proteins (NSPs) essential for the viral replication (Gorbalenya et al., 1989; Hegyi and Ziebuhr, 2002; Kiemer et al., 2004; Yan and Wu, 2021).

The essential role played by SARS-CoV-2 3CL^pro^ during the viral replication has encouraged the search for anti-Covid drugs targeting this protease. Numerous potent orthosteric inhibitors of 3CL^pro^, most of them of peptide-based or peptidomimetic nature, have been reported so far (Amin et al., 2021; Chia et al., 2021; Sabbah et al., 2021; Yan and Gao, 2021). These compounds have shown significant inhibitory activity, not only against the protease but also against the viral replication in cell cultures. Meanwhile, the crystal structures of 3CL^pro^ in complex with a myriad of inhibitors and compound fragments have been deposited in the Protein Data Bank (PDB) and provide useful structural information for the rational design of new drugs (Mengist et al., 2021). Some of these structures have revealed the existence of allosteric binding sites in the surface of 3CL^pro^, which can also be exploited to search for noncompetitive inhibitors (Douangamath et al., 2020; Gunther et al., 2021). More recently, the 3CL^pro^ peptidomimetic inhibitor PF-07321332 has shown promising results in phase I clinical trials, thus paving the way toward the discovery of an effective antiviral (Owen et al., 2021). All these results underscore the importance of 3CL^pro^ as an attractive drug target to combat Covid-19.

Even though diverse scaffolds of 3CL^pro^ inhibitors have been identified, D-peptides remain unexplored. These molecules are made up of D-amino acids, i.e., amino acids whose chiral Cα atoms have the opposite stereochemical configuration to that observed in the amino acids that commonly form the natural proteins (the L-amino acids). This structural feature endows D-peptides with key advantages over the L-peptides, such as higher stability to proteolysis, improved intestinal absorption upon oral administration, and low or missing immunogenicity. These properties, along with others shared with L-peptides, e.g., lower manufacturing costs and higher binding affinity and specificity for the target receptors in comparison with small molecules, make D-peptides attractive therapeutic agents (Wiesehan and Willbold, 2003; Funke and Willbold, 2009; Sun et al., 2012; Liu et al., 2016; Garton et al., 2018). Remarkably, α-helical D-peptides designed *in silico* were reported to block the binding of the SARS-CoV-2 spike protein receptor-binding domain (RBD) to the human angiotensin-converting enzyme 2 (ACE2), the molecule that mediates the virus internalization into human cells, thus leading to the inhibition of viral infection *in vitro* (Valiente et al., 2021). The previous results provide an excellent example of the use of D-peptides as promising anti-Covid drug candidates.

Structure-based virtual screening (SBVS) of diverse ligand databases, many of them containing drug repurposing candidates and natural products, has been extensively applied to identify potential 3CL^pro^ inhibitors (Wu C. et al., 2020; Chowdhury et al., 2020; Jukic et al., 2020; Meyer-Almes, 2020; Olubiyi et al., 2020; Selvaraj et al., 2020; Federico et al., 2021; Gogoi et al., 2021; Guedes et al., 2021; Kumar et al., 2021; Lokhande et al., 2021; Naik et al., 2021; Rajpoot et al., 2021; Rehman et al., 2021; Sisakht et al., 2021). In several cases, this approach has led to the successful identification of compounds displaying *in vitro* inhibitory activity against 3CL^pro^ (Ghahremanpour et al., 2020; Gupta et al., 2020; Jin et al., 2020; Li et al., 2020; Alves et al., 2021; Banerjee et al., 2021; Gunther et al., 2021; Guo et al., 2021; Gupta et al., 2021; Hamdy et al., 2021; Pathak et al., 2021; Yang et al., 2021). On the other hand, protein-peptide docking remains far more challenging compared to other small molecules due to the higher flexibility of peptides (Rentzsch and Renard, 2015; Ciemny et al., 2018; Hashemi et al., 2021). Nonetheless, at least one work has reported two L-pentapeptides as potential 3CL^pro^ inhibitors by screening a 70,000-peptide library (Porto, 2021), using AutoDock Vina for the docking simulations (Trott and Olson, 2010). Remarkably, AutoDock Vina outperformed other freely-available docking algorithms, such as AutoDock and ZDOCK (Chen and Weng, 2002; Morris et al., 2009), in a benchmark study that presented a pipeline for peptide SBVS (Ansar and Vetrivel, 2019).

Encouraged by the previous findings, this study presents D-peptides as 3CL^pro^ inhibitors. The computational workflow employed for D-peptide identification, which will be fully described in Materials and Methods, selects the best binders to the protease active site through SBVS and a series of rescoring steps combining Molecular Mechanics Generalized-Born Surface Area (MM-GBSA) free energy calculations (Gohlke et al., 2003; Gohlke and Case, 2004; Miller et al., 2012) and molecular dynamics (MD) simulations (Hou et al., 2011; Hernandez Gonzalez et al., 2021). The four top-ranked D-peptides were purchased and tested *in vitro* to evaluate their inhibitory activity against 3CL^pro^. Remarkably, all the tested D-peptides caused 3CL^pro^ inhibition at 20 μM during primary assays, resulting in up to 85% loss of proteolytic activity in certain cases. Therefore, the devised workflow led to promising results potentially extensible to broader applications related to protein inhibition.

## Materials and Methods

### Preparation of the Protein Structure for Virtual Screening and MD Simulations

The crystal structure of free 3CL^pro^ (PDB: 6Y2E, resolution 1.75 Å) (Zhang et al., 2020b) was chosen to conduct SBVSs and MD simulations with identified D-peptides. Protonation at pH 7.2 was performed using the PDB2PQR Web Server (https://server.poissonboltzmann.org/pdb2pqr) (Dolinsky et al., 2007). The protonated structure was then converted into the pdbqt file required for SBVS with the program *prepare_receptor4. py* of AutoDockTools 4 (Morris et al., 2009). MD simulations of the 3CL^pro^/peptide complexes were also performed using the predicted protonation states of the ionizable protein residues.

### Building an In-House Library of D-Tripeptides and D-Tetrapeptides

An in-house library of capped D-tripeptides and D-tetrapeptides (for brevity’s sake the term “capped” will be omitted hereinafter when referring to the D-peptides) was built using *tleap* of Amber20 (Case et al., 2020). Briefly, *tleap* was called inside three or four nested loops, depending on the peptide length, each iterating over all the different amino acids. For HIS, its two different neutral tautomers were considered, thus totalizing 21 residue types. The *sequence* command of *tleap* was employed to create each peptide, with acetyl (ACE) and N-methyl amide (NME) capping groups being added at the N- and C-termini, respectively. The default L configuration of the Cα atoms was then inverted to D configuration using the *flip* command of *tleap* (Case et al., 2020). Of note, the chiral centres of ILE and THR side-chains were not inverted, thus being modeled as D-allo-isoleucine and D-allo-threonine diastereomers, which will be referred to as ILE and THR hereinafter. The D-peptides were embedded into TIP3P octahedral solvation boxes, with edges spanning at least 10 Å from the solute surface, and counter-ions (Na^+^) were added to neutralize the system net charge. Topology and coordinate files for every solvated D-peptide were finally generated and saved for subsequent steps.

The systems were subjected to two rounds of energy minimization (EM) using *pmemd*. *MPI* of Amber20 in order to obtain a suitable conformation of each D-peptide in solution (Case et al., 2020). The first EM step consisted of 500 cycles of steepest descents (SD) followed by 500 cycles of conjugate gradient (CG) minimization, and both were carried out in the presence of harmonic restraints applied to the D-peptide heavy atoms (*k* = 10 kcal⋅mol^−1^⋅Å^2^). The second EM was performed with no harmonic restraints and, as before, involved 500 cycles of SD followed by 500 cycles of CG minimization. The energy-minimized D-peptides were then stripped off the solvent and counter-ions, saved as *pdb* files with *cpptraj* of Amber20 (Case et al., 2020), and converted into *pdbqt* files with *prepare_ligand4. py* of AutoDock Tools 4 (Morris et al., 2009). This step completed the preparation of the peptide library containing 9,261 D-tripeptides and 194,481 D-tetrapeptides. Libraries of larger peptides were not prepared, as docking algorithms tend to produce less accurate results for molecules bearing many freely-rotatable bonds (Rentzsch and Renard, 2015; Ciemny et al., 2018; Hashemi et al., 2021).

### Structure-based Virtual Screening

**FIGURE 1:**
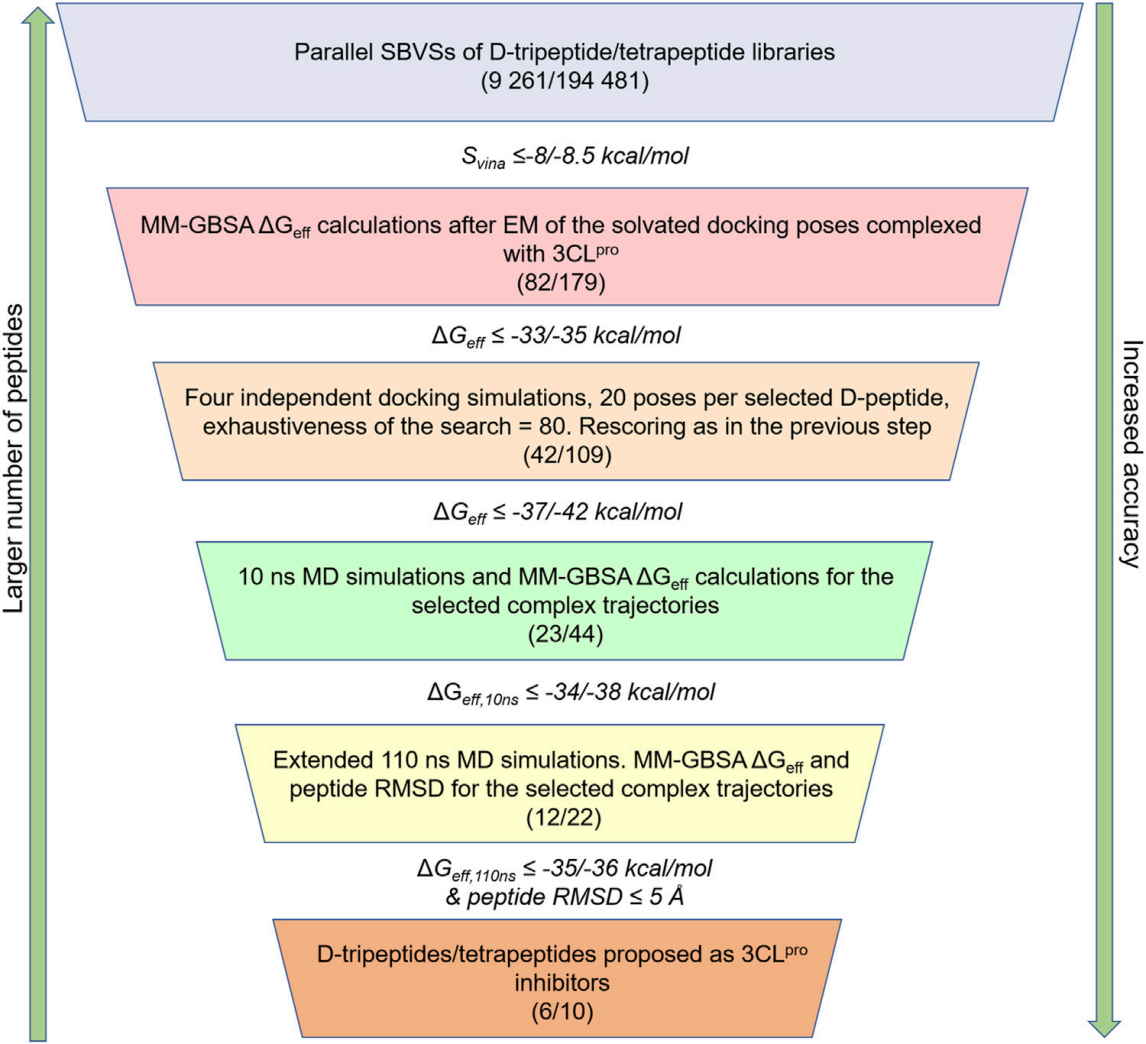
Workflow employed for the identification of potential D-peptides targeting the 3CL^pro^ active site. The number of D-tripeptides and D-tetrapeptides analyzed in parallel throughout the workflow is shown in parentheses, i.e., (number of D-tripeptides/number of D-tetrapeptides). The cut-offs used to select the peptides at every step are indicated as follows: cut-off for D-tripeptides/cut-off for D-tetrapeptides. These values were set on the basis of the results, so that a manageable number of D-peptides would be selected for the next step.

Despite 3CL^pro^ being a homodimer in solution, a monomer was chosen for SBVS and subsequent post-docking rescoring steps, as each active site in the functional homodimer is formed by residues belonging to an individual chain. A 19.5 Å × 18.0 Å x 22.0 Å box spanning the whole active site of 3CL^pro^ (PDB: 6Y2E) was then built using the Autodock/Vina plugin of Pymol (Supplementary Figure S1), and docking of D-peptides was performed with AutoDock Vina v1.12 (DeLano, 2002; Seeliger and de Groot, 2010; Trott and Olson, 2010). Default parameters, i.e., 9 poses per ligand, the exhaustiveness of the search equal to 8, and an energy difference of 3 kcal/mol between the best and worst poses were set for the docking simulations during SBVSs. D-tripeptide and D-tetrapeptide libraries were screened and ranked separately to reduce the impact of ligand-size bias (Chang et al., 2010). Based on the obtained AutoDock Vina scores (*S*_*vina*_), the 82 and 179 top-ranked D-tripeptides and D-tetrapeptides, respectively, were selected for subsequent rescoring steps (Figure 1).

### Post-docking Rescoring Steps

The selected D-tripeptides and D-tetrapeptides underwent in parallel a series of rescoring steps involving MM-GBSA free energy calculations (Gohlke et al., 2003; Gohlke and Case, 2004; Miller et al., 2012; Case et al., 2020), docking with increased exhaustiveness of the search and MD simulations (Figure 1). First, each of the nine docking poses per selected D-peptide was rescored based on their MM-GBSA effective free energies (Δ*G*_*eff*_), calculated for the energy-minimized solvated complexes. The D-peptides were then re-ranked according to the Δ*G*_*eff*_ values of their respective lowest-energy poses. The best D-peptides were then docked again into the 3CL^pro^ active site with AutoDock Vina. For this step, the exhaustiveness of the search was increased to 80, and four independent and randomly-initialized docking simulations, each generating 20 different poses (80 poses in total), were run for every D-peptide. As before, lowest-energy poses were identified by rescoring the 80 docking poses per ligand using their corresponding MM-GBSA Δ*G*_*eff*_ values after EM of the 3CL^pro^/peptide complexes. Finally, 10 ns of MD simulations, subsequently extended to 110 ns for the best candidates, were conducted, and average MM-GBSA Δ*G*_*eff*_ values were calculated for the generated trajectories (Δ*G*_*eff,10ns*_ and Δ*G*_*eff,110ns*_, respectively). The D-peptides with average Δ*G*_*eff,110ns*_ and Root Mean Squared Deviation (RMSD) values (see *Trajectory Analyses* section) lower than the indicated cut-offs were proposed as potential 3CL^pro^ inhibitors (Figure 1).

### MD Simulations Setup

The 3CL^pro^/peptide complexes were parametrized with Amber ff14SB force-field (Maier et al., 2015) and embedded in octahedral simulation boxes with edges spanning, at least, 10 Å away from the solute surface, filled with TIP3P waters (Price and Brooks, 2004) and sufficient Na^+^ counter-ions to neutralize the systems (Supplementary Table S1) (Li et al., 2015). All previous steps were carried out with *tleap* of Amber20 (Case et al., 2020). Again, as individual chains form the two active sites of the 3CL^pro^ homodimer, MD simulations were carried out with monomeric 3CL^pro^ in complex with the selected D-peptides. This approach was deemed sufficiently accurate to assess the complex stability and significantly reduced the computational demand by decreasing the size of the simulated systems. The solvated complexes underwent two consecutive EMs identical to those conducted during the preparation of the D-peptide libraries. Each energy-minimized system was equilibrated prior to the productive run through a 1 ns NVT heating using a linear temperature gradient from 10 to 298.15 K, followed by a 1 ns NPT equilibration at *p* = 1 bar and *T* = 298.15 K. Both equilibration steps were carried out in the presence of harmonic restraints (*k* = 10 kcal⋅mol^−1^⋅Å^−2^) applied to the complex heavy atoms. Then, four consecutive 1 ns MD simulations in the NPT ensemble, in which the harmonic constant was lowered from 8 to 2 kcal mol^−1^∙Å^−2^ in 2 kcal mol^−1^∙Å^−2^ strides, were carried out. Subsequently, 10 ns NVT MD simulations, extended to 110 ns for several systems showing favorable Δ*G*_*eff,10ns*_ values, were conducted. Finally, the MD simulations corresponding to the four experimentally tested D-peptides in complex with 3CL^pro^ were extended to 1 µs. Replicate 1 μs MD simulations for these systems were also performed by subjecting the last frame of the respective 110 ns trajectories to EM, heating using randomly-initialized atomic velocities drawn from a Maxwell-Boltzmann distribution, and NPT equilibration steps, as described hereinbefore.

The program *pmemd.cuda* of Amber20 was used to run all MD simulations (Salomon-Ferrer et al., 2013; Case et al., 2020). Periodic boundary conditions were set during both EM and MD simulations, and long-range electrostatic interactions (for distances >9 Å) were handled with the Particle Mesh Ewald (PME) algorithm (Darden et al., 1993). Temperature control was carried out using the Berendsen weak coupling algorithm (Berendsen et al., 1984) during heating and the Langevin thermostat (Schneider and Stoll, 1978), with a collision frequency of 2 ps^−1^, during both NPT equilibration and NVT production runs. The Berendsen barostat (Berendsen et al., 1984), with a relaxation time of 2 ps, was employed to control the pressure during NPT equilibrations. Covalent bonds involving hydrogen atoms of the solute and water molecules were constrained with the SHAKE (Ryckaert et al., 1977) and SETTLE (Miyamoto and Kollman, 1992) algorithms. The equation of motion of the simulated systems was integrated using the *leap-frog* algorithm with a timestep of 2 fs (Case et al., 2020).

### MM-GBSA Free Energy Calculations

MM-GBSA free energy calculations were conducted with the *MMPBSA.py* program of Amber20 (Gohlke et al., 2003; Gohlke and Case, 2004; Miller et al., 2012; Case et al., 2020). The single trajectory approach, in which the free ligand and the free receptor trajectories are extracted from that of the complex, was adopted in all cases after stripping off the solvent and ions (Miller et al., 2012). The GB-neck2 implicit solvation model was employed to determine the polar solvation free energy component (Nguyen et al., 2013; Case et al., 2020), as it yielded a good correlation with experimental results in a set of protein-peptide complexes (Hernandez Gonzalez et al., 2017). Moreover, the calculations were performed using the *mbondi3* set of atomic radii, a salt concentration of 0.1 M and external and internal dielectric constants of 80 and 1, respectively. The surface tension and the offset values were set to 0.0072 kcal⋅mol^−1^⋅Å^−2^ and zero, respectively, in order to estimate the nonpolar free-energy component from the variation of the solvent accessible surface areas (SASAs) of the interacting molecules, i.e., 3CL^pro^ and D-peptides (Case et al., 2020). In turn, SASA values were obtained with the Linear Combination of Pair-wise Overlaps (LCPO) algorithm included in Amber20 suite (Weiser et al., 1999; Case et al., 2020) using a probe radius of 1.4 Å (Connolly, 1983). As mentioned before, the Δ*G*_*eff,10ns*_ and Δ*G*_*eff,110ns*_ values obtained from the 10 and 110 ns MD simulations after discarding the first 2 and 40 ns, respectively, allowed us to select the most promising D-peptides at the two last steps of the workflow shown in Figure 1.

### Principal Component Analysis

Principal component analysis (PCA) (Amadei et al., 1993) was carried out for the 1 μs trajectories of 3CL^pro^ in complex with the experimentally-tested D-peptides. Using this technique, we sought to reduce the phase space dimensionality by projecting the system’s motion along the two eigenvectors, known as principal components (PCs), of the highest variance (=largest eigenvalues), as our main interest here was to identify the different conformations of the D-peptides in the 3CL^pro^ active site during the long MD simulations. PCA was performed only for the Cα atoms of the former molecules. The replicate 1 μs MD simulations of each system were concatenated and fitted using *cpptraj* of Amber 20 (Case et al., 2020). Trajectory fitting was carried out concerning the 3CL^pro^ backbone atoms belonging to the chymotrypsin-like (ChT-like) domains, i.e., domains I and II, residues 8 to 183 (Tahir Ul Qamar et al., 2020), which contain the active site. This step ensured that the displacements of the D-peptide Cα atoms during the long MD simulations were measured relative to the enzyme’s active site and eliminated the influence of domain III motions during fitting. The program *gmx covar* of Gromacs v.5.1.4 (Abraham et al., 2015) was employed to calculate the covariance matrices of the D-peptide Cα positions along the fitted trajectories and their corresponding sets of eigenvalues and eigenvectors. Two-dimensional (2D) projections of the trajectories onto the first two eigenvectors (PC1 and PC2) were obtained with *gmx anaeig* of Gromacs v5.1.4.

PCA was combined with free energy landscape (FEL) visualization and clustering to determine central structures for the different conformations sampled during the MD simulations (Papaleo et al., 2009). FELs were obtained from the 2D projections and depicted as heatmaps by discretizing the 2D phase space into 700 square bins of equal size and counting the number of points within each. The free energy value corresponding to bin *i* (Δ*G*_*i*_) was then calculated from the probability of finding the system into that bin (*p*_*i*_) using the equation:

$$\Delta G_{i}=-RTln\left(p_{i}\right)$$ (1)

where *R* is the gas constant and *T*, the temperature (298.15 K).

Finally, the trajectories were split using the K-means algorithm with random initial seeds, implemented as an option of the *cluster* command of *cpptraj* (Case et al., 2020), by using the PC1 and PC2 values as a metric. The number of clusters in each case was set to the main FEL basins observed in the corresponding heatmap. Each newly generated trajectory was subsequently clustered to determine its central structure through RMSD clustering (see next section for details).

### Trajectory Analyses

The 110 ns MD simulations were clustered in order to select the central structure of each analyzed 3CL^pro^/peptide complex. This step was performed with the *cluster* command of *cpptraj* using the average linkage algorithm (Shao et al., 2007; Case et al., 2020). The RMSD for the heavy atoms of the peptide and 3CL^pro^ residues lying within a 4 Å cut-off was chosen as a metric for clustering the trajectories. This procedure was also applied to determine the central structures corresponding to the main energy minima observed in the PC1 vs PC2 projections of the complexes subjected to PCA. The main central structures were selected for structural representation using Pymol 2.1.0 (DeLano, 2002). RMSD values for the D-peptide heavy atoms along the 110 ns trajectories were calculated with *rms* of *cpptraj* after fitting all frames with respect to the 3CL^pro^ backbone atoms belonging to the ChT-like domains in the corresponding starting structures (*t* = 0). These RMSD values were averaged during the last 20 ns of the trajectories to assess whether the binding modes of the D-peptides sampled at the end of the MD simulations deviated significantly from those of the starting structures. Root mean square fluctuations (RMSFs) were calculated with the *rmsf* command of *cpptraj* (Case et al., 2020). Finally, hydrogen bonds (H-bonds) formed at the complex interfaces during the MD simulations were determined with *hbond* command of *cpptraj* (Case et al., 2020), using the following geometric criteria: a donor-acceptor distance ≤3.5 Å and a donor-H-acceptor angle ≥120°.

### D-Peptide Synthesis

Synthetic D-enantiomeric peptides used in this study (4P1, 4P2, 4P3, and 4P4) were synthesized by Genscript (Leiden, NL), with a purity of ≥90%. The D-peptides were acetylated at the N-terminus and methylated at the C-terminus.

### Cloning, Expression, and Purification of SARS-CoV-2 3CL^pro^

The codon-optimized cDNA encoding SARS-CoV-2 3CL^pro^ (Uniprot entry: P0DTD1, virus strain: hCoV-19/Wuhan/WIV04/2019) was synthesized and implemented in the ampicillin-resistant vector pGEX-6P-3 (BioCat GmbH, Heidelberg, Germany). The construct contains an N-terminal GST-tag and a PreScission protease cleavage site (LEFLFQGP). Expression and purification were performed as described before (Eberle et al., 2021).

### Primary 3CL^pro^ Enzymatic Inhibition Assay

All measurements were performed in triplicate in 20 mM Tris pH 7.2, 200 mM NaCl, 1 mM EDTA, and 1 mM TCEP as described previously (Zhang et al., 2020a; Zhang et al., 2020b; Ma et al., 2020; Eberle et al., 2021). 20 µM of the peptides (4P1, 4P2, 4P3, and 4P4) were pipetted into a Corning 96-Well plate (Sigma Aldrich), 3CL^pro^ was added to a final concentration of 500 nM, and the mixture was incubated for 30 min. Subsequently, the enzymatic reaction was initiated by adding the fluorogenic substrate DABCYL-KTSAVLQ↓SGFRKME-EDANS (Bachem, Switzerland) to a final concentration of 50 µM. The gradual release of fluorescent 5-((2-Aminoethyl)amino)naphthalene-1-sulfonic acid (EDANS) was monitored for 30 min with 60 s intervals. The excitation and emission wavelengths were 360 and 460 nm, respectively, using an Infinite 200 PRO plate reader (Tecan, Männedorf, Switzerland). The temperature was set to 37°C. The results are shown as mean value ±standard deviation (STD).

### Statistical Analyses

Block averaging was conducted using *gmx analyze* of Gromacs v.5.1.4 (Hess, 2002; Abraham et al., 2015) to estimate standard errors of the mean (SEMs) from time-dependent values collected from MD simulations, such as the reported Δ*G*_*eff,110ns*_ and RMSD mean values. On the other hand, the statistical significance of the residual activity mean values’ differences was performed with GraphPad Prism software version 8 (GraphPad, 2018) and was assessed with one-way analyses of variance (ANOVA), followed by Tukeys’ multiple comparison test. Significant differences were considered at *p* < 0.01 (**) and *p* < 0.001 (***).

## Results

### D-Peptides Predicted as 3CL^pro^ Inhibitors

**FIGURE 2:**
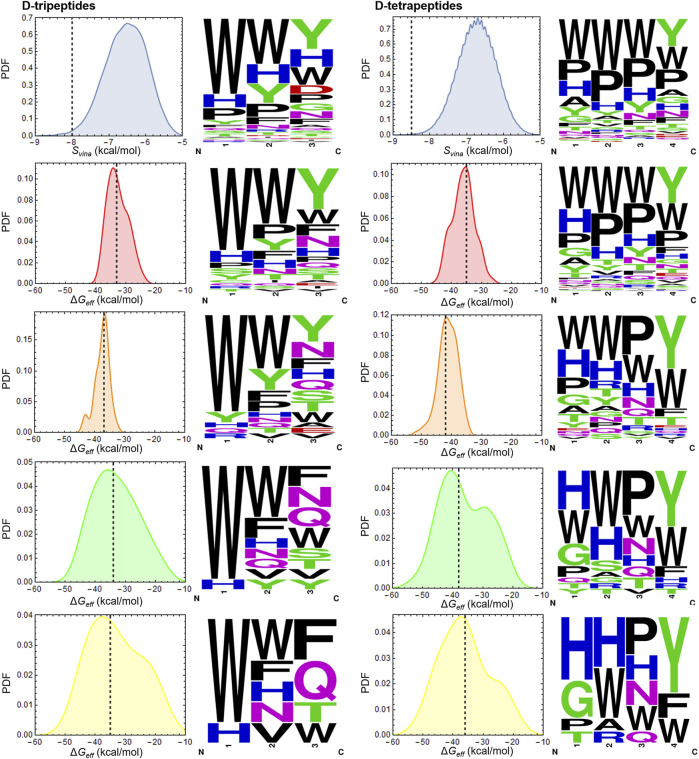
Energy distributions and D-peptide sequences at every step of the *in silico* workflow. Each histogram is colored as the corresponding step of the workflow shown in Figure 1. Dashed lines indicate the cut-off values employed to select the best candidates at every step. Peptides with *S*_*vina*_/Δ*G*_*eff*_ values to the left of the dashed lines were selected. PDF is the abbreviation for probability density function. Sequence logos of the D-peptides identified at every step are shown to the right of the corresponding histograms. Letter sizes are proportional to the frequency of occurrence of the indicated residues in each position. N and C below the graphs stand for the N- and C- termini, respectively. Sequence logos were generated at https://weblogo.berkeley.edu/logo.cgi. A D-tetrapeptide was excluded as potential 3CL^pro^ inhibitor after filtering according to the RMSD value in the last rescoring step (Figure 1, not shown). The last sequence logo of D-tetrapeptides does not contain the excluded peptide.

The D-peptides proposed as potential 3CL^pro^ inhibitors were selected through a workflow involving SBVS and several rescoring steps (Figures 1, 2). The top-ranked D-peptides, according to the *S*_*vina*_ values, were made up mainly of aromatic and hydrophobic residues, with TRP being the residue most frequently found in all positions except the C-terminus, in which TYR was the most abundant (Figure 2). Certain regions of 3CL^pro^ active site, such as the S2 pocket, are hydrophobic (Jin et al., 2020), which can favor the binding of peptides containing the aforementioned residues. However, the already known bias of AutoDock Vina and other docking algorithms toward larger compounds can also be at play here (Pan et al., 2003; Chang et al., 2010). The subsequent rescoring steps aimed to correct this bias and enrich selected D-peptides’ lists with accurate hits. In fact, it can be observed from the sequence logos, shown in Figure 2, that even though TRP was still prevalent at different positions of the D-peptides, other residues became progressively more abundant throughout the workflow steps, which was particularly apparent for D-tetrapeptides, in which HIS and PRO were found to be predominant in positions 1 to 3 after completing the workflow (Figure 2).

Apart from HIS, other residues with polar side-chains, such as ASN, GLN, THR, and ARG, occurred more frequently among the D-peptides selected after the last rescoring step than among those selected from the SBVS. Consequently, the D-peptides prioritized after the workflow completion were, on average, smaller in size than those ranked in the top positions by the docking algorithm. Overall, the rescoring steps tended to upweight the occurrence of intermolecular H-bonds mediated by the side-chains of D-peptide residues at the expense of ligand size. These results, in turn, suggest a reduced impact of ligand-size bias on the final set of chosen D-peptides. The D-tripeptides and D-tetrapeptides identified as potential 3CL^pro^ inhibitors are shown in Table 1. All these D-peptides fulfilled the conditions set throughout the workflow steps. They showed a good affinity for the enzyme and stability of the initial docking pose during the MD simulations, measured in terms of Δ*G*_*eff,110ns*_ and peptide RMSD mean values, respectively (Table 1 and Supplementary Figures S2, S3).

**TABLE 1:** D-peptides identified *in silico* as potential 3CL^pro^ inhibitors.

Peptide ID	Peptide sequence^a^	Δ*G*_*eff,110ns*_ (kcal/mol)^b^	RMSD (Å)^c^
D-tripeptides			
3P1	ACE-TRP-TRP-THR-NME	−44.2 ± 0.5	2.58 ± 0.03
3P2	ACE-TRP-ASN-PHE-NME	−43.8 ± 1.0	2.6 ± 0.3
3P3	ACE-TRP-PHE-GLN-NME	−40.4 ± 2.0	4.22 ± 0.05
3P4	ACE-TRP-VAL-PHE-NME	−40.3 ± 1.3	2.3 ± 0.3
3P5	ACE-TRP-TRP-GLN-NME	−36.5 ± 0.6	4.43 ± 0.04
3P6	ACE-HIE-HID-TRP-NME	−35.6 ± 1.3	1.22 ± 0.06
D-tetrapeptides			
4P1	ACE-GLY-TRP-ASN-TYR-NME	−50.2 ± 0.9	3.60 ± 0.04
4P2	ACE-GLY-TRP-HIE-TRP-NME	−45.6 ± 0.7	3.0 ± 0.9
4P3	ACE-HIE-ALA-PRO-TRP-NME	−44.9 ± 0.9	1.17 ± 0.05
4P4	ACE-HIE-HIE-PRO-TYR-NME	−44.9 ± 1.5	2.70 ± 0.09
4P5	ACE-THR-HIE-TRP-TYR-NME	−44.5 ± 2.0	3.574 ± 0.08
4P6	ACE-HIE-HIE-ASN-TYR-NME	−42.8 ± 0.9	3.20 ± 0.04
4P7	ACE-HIE-TRP-PRO-PHE-NME	−39.2 ± 0.7	3.33 ± 0.06
4P8	ACE-HIE-HIE-HID-TYR-NME	−38.8 ± 0.7	3.5 ± 0.2
4P9	ACE-PRO-TRP-GLN-PHE-NME	−38.7 ± 0.5	2.20 ± 0.02
4P10	ACE-GLY-ARG-TRP-TYR-NME	−37.5 ± 2.0	3.36 ± 0.09

a Residues are shown in three-letter code and separated by hyphens. ACE and NME are the N- and C-terminal caps added to the D-peptides. HIE and HID are HIS tautomers.

b MM-GBSA average effective free energies calculated over the last 70 ns of each 110 ns MD trajectory ± SEMs estimated through block averaging. See instantaneous ΔG_eff_ values vs time plots for every system along their respective 110 ns MD simulations in Supplementary Figure S2.

c Mean RMSD values for the peptide heavy atoms with respect to the starting structure (*t =* 0) calculated over the last 20 ns of each 110 ns MD trajectory ± standard errors of the mean estimated through block averaging. See RMSD values vs time plots for all systems along their respective 110 ns MD simulation in Supplementary Figure S3.

### Structural Features of the Predicted 3CL^pro^/D-peptide Interfaces

**FIGURE 3:**
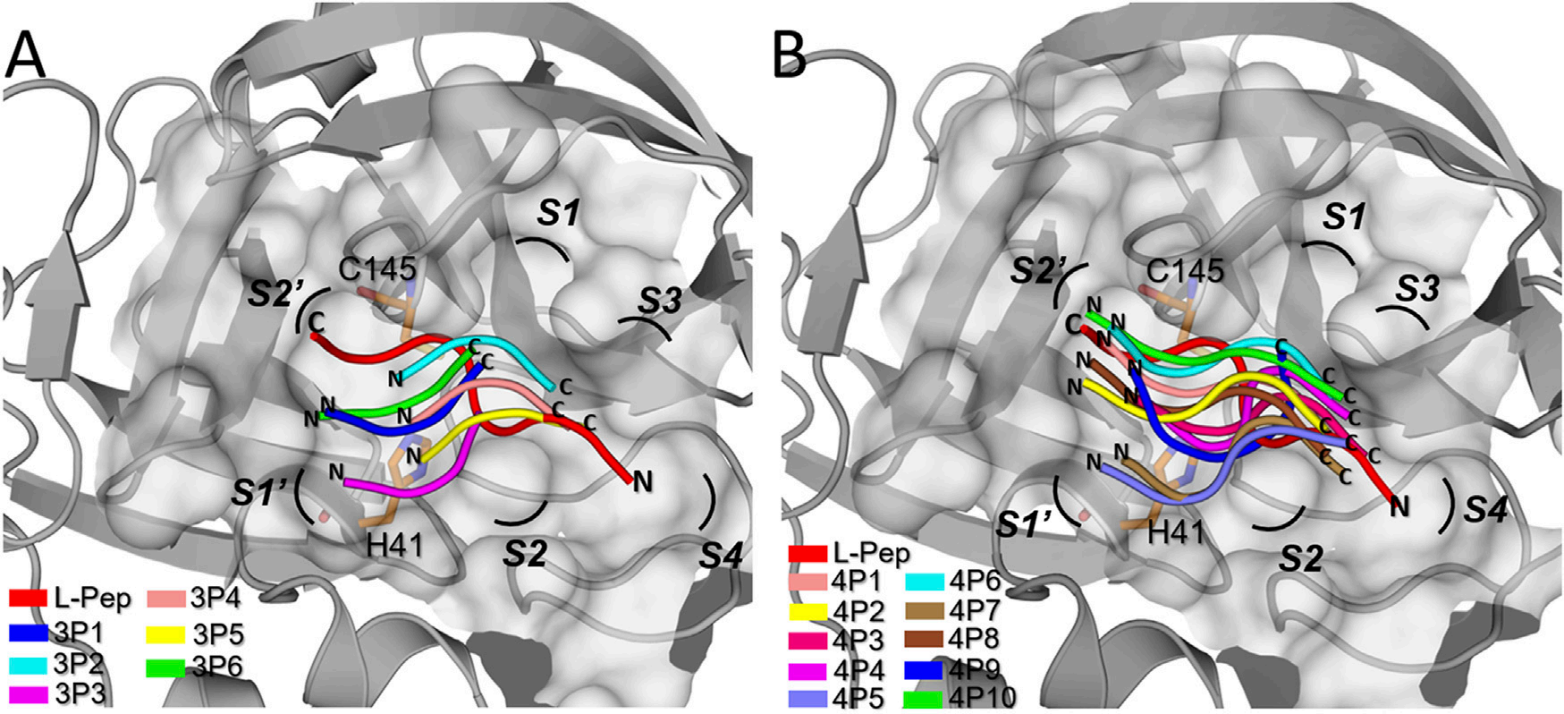
Accommodation of the D-peptide backbones into the active site of 3CL^pro^. Superimposed central structures of 3CL^pro^ in complex with the **(A)** D-tripeptides and **(B)** D-tetrapeptides proposed as potential inhibitors (Table 1), determined from the respective 110 ns MD simulations. In both cases, the crystal structure of 3CL^pro^ (PDB: 6Y2E) is depicted in gray and the catalytic residues, H41 and C145, are shown as orange sticks. The protease active site is represented as a gray surface. The D-peptides of the structurally-aligned complexes are colored differently (see legends). The 3CL^pro^ conformation of each central structure was omitted for clarity’s sake. For comparison purposes, each panel shows the conformation of the L-peptide VTLQSK (L-Pep) into SARS-CoV-2 3CL^pro^, obtained through structural alignment between the former protease and the crystal structure of SARS-CoV 3CL^pro^ (PDB: 5B6O) (Muramatsu, et al., 2016). L-Pep corresponds to the C-terminal portion of a neighboring SARS-CoV 3CL^pro^ chain that inserts in a substrate-like conformation into the protease active site. Letters N and C indicate the N- and C-termini of the peptides, respectively. The 3CL^pro^subsites S2′ to S4 are labeled accordingly.

The central structures of the selected D-peptides (Table 1) in complex with 3CL^pro^ obtained after clustering the respective 110 ns MD simulations are shown in cartoon representation in Figure 3. Moreover, for comparison purposes, the L-peptide VTLQSK (L-Pep) is depicted at the 3CL^pro^ active site (Figure 3). L-Pep corresponds to the C-terminus of the homologue SARS-CoV 3CL^pro^ and inserts into the active site of a neighboring protease chain in the PDB structure 5B6O (Muramatsu et al., 2016), thus allowing the template-based modeling of the SARS-CoV-2 3CL^pro^/L-Pep complex. Of note, the backbones of all the analyzed D-peptides adopt an orientation opposite (=retro-binding) to that of L-Pep (Figures 3A,B). As we will show below in more detail, the retro-binding enables the formation of key interactions between the active site residues and the backbone atoms of the D-peptide present at the 3CL^pro^/L-Pep interface.

It can be seen that there is a partial overlap between the L-Pep and D-peptide backbones, especially involving the N- and C-terminal portions of L-pep and D-tetrapeptides, respectively (Figure 3). Therefore, several key interactions mediated by the peptide backbones and neighboring residues on the S side of the 3CL^pro^ active site are expected to be preserved in the predicted complexes. The D-tetrapeptide backbones span a larger stretch of the 3CL^pro^ active site when compared to the D-tripeptides and resemble, to a greater extent, the overall accommodation of L-Pep, which could explain, in turn, the generally more favorable free energy values obtained for the D-tetrapeptides in complex with 3CL^pro^ (Table 1). On the other hand, the backbone accommodation was more divergent in the N terminal regions of the D-peptides, not only relative to L-pep but among themselves (Figure 3). This might arise from the plasticity of the 3CL^pro^ active site (Kneller et al., 2020) and the inability of the small D-peptides to satisfy interactions equivalent to those observed for L-peptides.

The number of intermolecular H-bonds formed along the MD simulations can be used to indicate complex stability (Lokhande et al., 2021). Therefore, we decided to calculate the time profiles of such interactions in the predicted 3CL^pro^/D-peptide complexes (Supplementary Figure S4). The graphs show that all the D-peptides in Table 1 formed several H-bonds with 3CL^pro^ active site residues during the 110 ns MD simulations of the complexes (Supplementary Figure S4). The average number of intermolecular H-bonds ranged from 3 to 8, depending on the complex. This result underscores the good complementarity of the identified D-peptides to the 3CL^pro^ active site.

**FIGURE 4:**
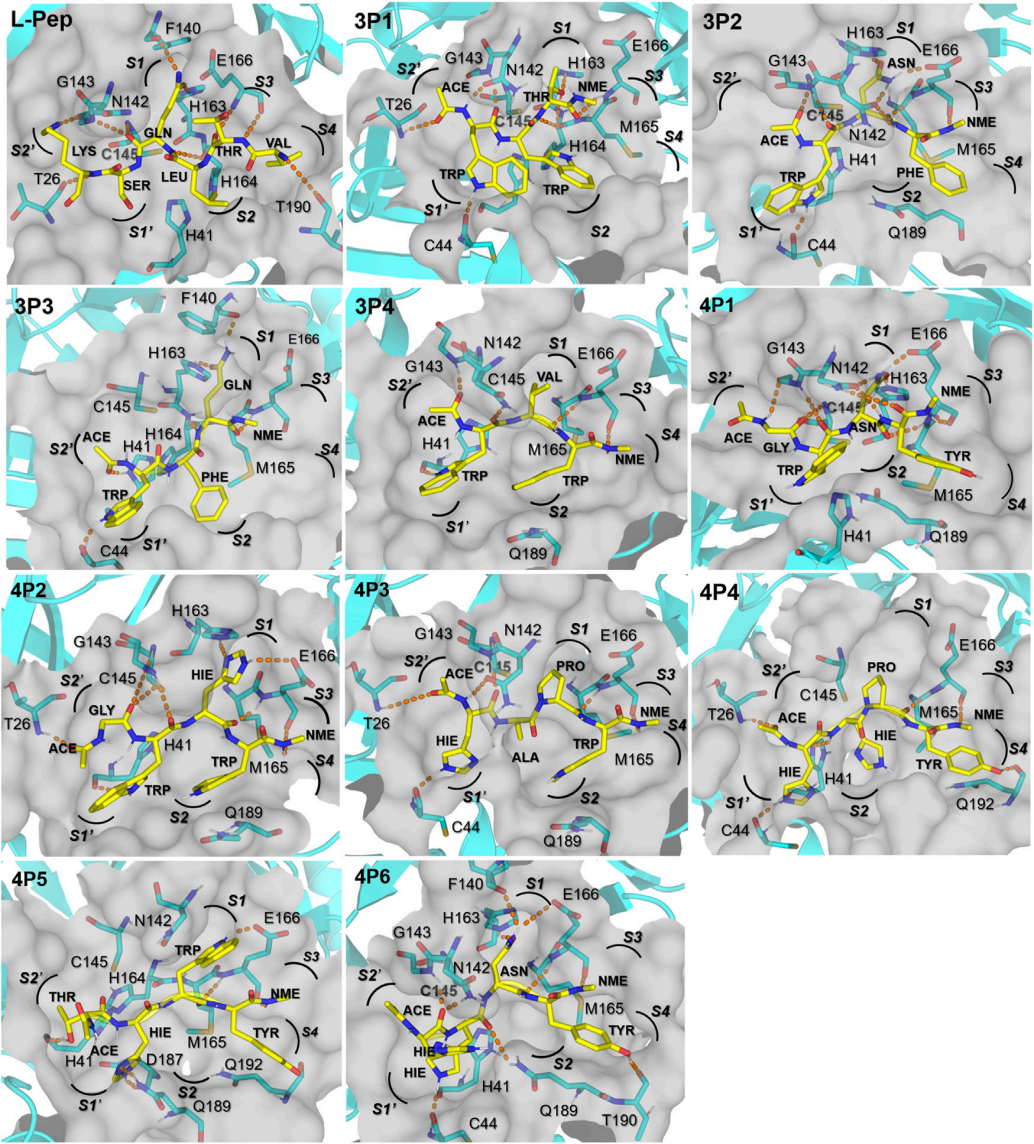
Structural representation of top-ranked D-tripeptides and D-tetrapeptides in complex with 3CL^pro^. All D-peptides are shown as yellow sticks and their residues are labeled in bold and in the three-letter code. 3CL^pro^ residues forming H-bonds with the peptides plus the catalytic residues H41 and C145 are labeled and represented as cyan sticks. The 3CL^pro^ active site cavity is depicted as a transparent gray surface. H-bonds between the D-peptides and the 3CL^pro^ residues with occupancies >25% during the respective 110 ns MD simulations are displayed as orange dashed lines. Subsites S4 to S2′ are labeled in bold and italic. Structural representations of the remaining D-peptides included in Table 1 can be found in Supplementary Figure S5.

The analysis of the interactions occurring at the 3CL^pro^/L-Pep complex provides valuable information to study the 3CL^pro^/D-peptide interfaces. Therefore, a detailed structural representation of this complex was included in Figure 4. L-Pep extends along with the S4 to S2′ subsites of the enzyme, displaying the N-terminal VAL residue at position P4 and the C-terminal LYS at P2’. THR at P3 is exposed mainly to the solvent, whereas LEU and GLN at P2 and P1, respectively, insert into well-defined pockets. As can be observed, several amide nitrogen (N) and carbonyl oxygen (O) atoms of L-Pep backbone engage in H-bond formation with 3CL^pro^ residues, e.g., VAL(N)-T190(O), THR(N)-E166(O), THR(O)-E166(N), GLN(N)-H164(O), GLN(O)-C145(N), GLN(O)-G143(N), and LYS(N)-T26(O) (Figure 4). Of note, the amide oxygen (OE1) of L-Pep GLN side-chain forms a key H-bond with the protonated N atom of H163 imidazole ring H163(NE2), which helps explain the preference of 3CL^pro^ for the former residue at P1 (Singh et al., 2020). Other H-bonds mediated by the side-chains of L-Pep residues are GLN(NE2)-F140(O) and LYS(NZ)-G143(O) at the S1 and S2′ subsites, respectively (Figure 4).

Structural representations of the interfaces of 3CL^pro^ in complex with the top-ranked D-tripeptides (Table 1) are shown in Figure 4. For brevity’s sake, only the complexes having Δ*G*_*eff*_ *,*_*110ns*_ values < −40 kcal/mol were included in the figure (Table 1). The backbone polar atoms of the D-tripeptides form a network of H-bonds with 3CL^pro^ residues, some of them equivalent to those observed at the interface of the L-Pep complex. For example, the H-bond involving an O atom of the D-tripeptides and E166(N) occurs in all the analyzed interfaces. An additional H-bond between a D-tripeptide N atom and E166(O) was found in the 3P2 and 3P4 complexes (Figure 4). H-bonds mediated by G143(N) and C145(N) and a D-tripeptide backbone O atom also occur in most depicted complexes. Differently from the L-Pep complex, the amide group of N142 can form H-bonds with polar atoms of 3P1, 3P2, and 3P4 backbone, thus providing additional stabilizing interactions. This result underscores that the flexible side-chain of N142 can adopt conformations in solution that facilitate transient H-bonding to the ligands.

Three top-ranked D-tripeptides (3P1, 3P3, and 3P4) possess aromatic residues TRP or PHE inserted into the S2 pocket of 3CL^pro^, which prefers hydrophobic residues (Chuck et al., 2010; Rut et al., 2021), whereas PHE of 3P2 occupies the S4 subsite (Figure 4). The S1 subsite accommodates the D-tripeptide residues THR, ASN, GLN, and VAL. Of note, ASN(OD1) and GLN(OE1) of 3P2 and 3P3, respectively, form the key H-bond with H163(NE2) observed for L-Pep GLN (Figure 4). The ASN side-chain can mimic the interactions established by the L-Pep GLN amide group because the backbone of 3P2 leans toward the entrance of the S1 pocket, thus shortening the distance to reach the bottom of this subsite (Figure 4). Like L-Pep GLN, the amide group of 3P2 ASN and 3P3 GLN can form additional H-bonds at S1, such as ASN(ND2)/GLN(NE2)-F140(O) or ASN(ND2)/GLN(NE2)-E166(OE1,2). Interestingly, the hydroxyl oxygen of 3P1 THR, THR (OG1), is also able to interact with H163(NE2), which could explain the favorable Δ*G*_*eff,110ns*_ value obtained for this D-peptide (Table 1) despite not bearing GLN or ASN at P1. Finally, it is worth noting that all the D-tripeptides proposed as 3CL^pro^ inhibitors possess TRP at the N-terminus, except for 3P6 that contains HIE (Table 1). Our results indicate that TRP accommodates favorably at the S1′ pocket, and the nitrogen of the indole group atom (NE1) can form H-bonds with C44(O)/H41(O) (Figure 4).

As done for the D-tripeptides, the interfaces of the top-ranked D-tetrapeptides (Δ*G*_*eff,110ns*_ < −40 kcal/mol, Table 1) in complex with 3CL^pro^ are depicted in Figure 4. The backbone of the selected D-tetrapeptides span the 3CL^pro^ active site from the S4 to the S2′ subsites and establish several polar interactions with the neighboring residues. Key H-bonds with E166(N) and E166(O), observed in the 3CL^pro^/L-Pep complex, occur in all the analyzed D-tetrapeptide complexes. Other H-bonds, such as those involving C145(N), G143(N), the N142 side-chain amide group, T26(N) and Q192(NE2), can be found at various interfaces (Figure 4).

Four out of the six top-ranked D-tetrapeptides, i.e., 4P1, 4P4, 4P5, and 4P6, contain TYR at the C-terminus, the most abundant residue occurring at this position in the whole set of identified D-tetrapeptides (Figure 2 and Table 1). The predicted structures suggest that TYR accommodates at the S4 subsite, where the aromatic ring sits on the pocket base, mainly formed by M165 and Q192, and the side-chain hydroxyl oxygen, TYR (OH), can form H-bonds with T190(O)/Q192(O) (Figure 4). The other two D-tetrapeptides, 4P2 and 4P3, insert their C-terminal TRP residues into the S2 pocket of 3CL^pro^. The latter D-peptide also accommodates its second residue, ALA, on the opposite side of the same pocket. Likewise, 4P4 can accommodate its second residue, HIE2, at the S2 pocket (Figure 4). A closer look at their sequences and the predicted structures of their complexes with 3CL^pro^ reveals that PRO at P1 of 4P3 and 4P4 bends the D-peptide backbones in a way that makes it feasible for upstream residues to interact with S2. Like PRO, ASN was found at the S1 pocket of 3CL^pro^ in complex with two other D-tetrapeptides, i.e., 4P1 and 4P6 (Figure 4). However, unlike the former residue, which leaves the S1 pocket largely unoccupied, ASN can form H-bonds equivalent to L-Pep GLN. At the same position, 4P2 HIE interacts with H163(NE2) and E166(OE1,2). Of note, HIS is the second most favorable residue at P1 according to substrate specificity profiling conducted for SARS-CoV 3CL^pro^ (Chuck et al., 2010). However, to reach the bottom of the S1 pocket, HIS, like ASN, requires a backbone accommodation closer to the pocket entrance, which is accessible to the D-peptides according to our predictions. The specificity for HIS at P1 in L-peptides has been explained by proposing that this residue interacts with the N142 side-chain (Chuck et al., 2010). TRP was also found at the S1 subsite of the 3CL^pro^/4P5 complex (Figure 4). In this case, the residue does not penetrate deeply into the subsite but forms the H-bond TRP(NE1)-E166(OE1,2).

HIE and TRP, in that order, are the most abundant residues of the top-ranked D-tetrapeptides placed at the S1′ subsite (Figure 4). HIE (NE2) is capable of forming H-bonds with C44(O) at the interfaces of 3CL^pro^ in complex with 4P3, 4P4, and 4P6 or with Q189(NE2) and D187(O) at the interface of the 3CL^pro^/4P5 complex. Interestingly, the latter D-peptide is the only one bearing an N-terminal THR residue, which forms H-bonds with H41(O). In this case, the ACE cap is sticking out to the solvent instead of lying at the S2′ subsite, as in the remaining complexes of the identified D-tetrapeptides (Figure 4).

### *In vitro* Inhibitory Activity of the Four Top-Ranked D-Tetrapeptides Against 3CL^pro^

The energetic and structural analyses presented in the previous sections demonstrated that the selected D-tetrapeptides displayed, in general, more favorable free energy values and better complementarity with the 3CL^pro^ active site than D-tripeptides (Table 1 and Figures 3, 4). Therefore, to assess the validity of the computational workflow for D-peptide identification, we decided to test *in vitro* the inhibitory activity of the four top-ranked D-tetrapeptides, 4P1 to 4P4 (Table 1).

**FIGURE 5:**
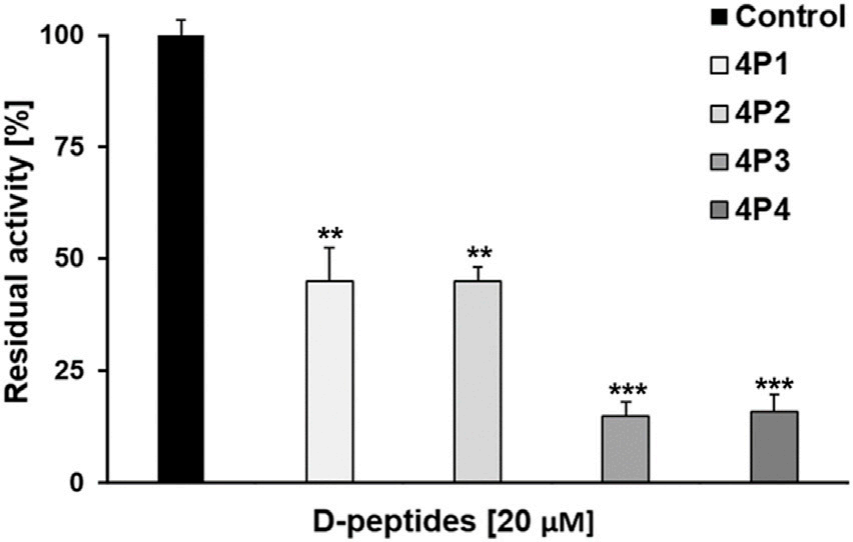
Preliminary inhibition tests of 4P1-4 against 3CL^pro^. 4P1 and 4P2 inhibit the virus protease activity by more than 60%. 4P3 and 4P4 inhibit the virus protease activity more than 80%. Data shown are the mean ± STD from three independent measurements (n = 3). Asterisks mean that the data differs from the control (0 µM inhibitor) significantly at *p* < 0.01 (**) and *p* < 0.001 (***), level according to ANOVA and Tukey’s test.

The results of the primary screening are shown in Figure 5. All the D-tetrapeptides, tested at a final concentration of 20 μM, significantly reduced the activity of 3CL^pro^ relative to the control assay. In fact, the percentage of residual activity of the protease dropped below 20% upon incubation with 4P3 and 4P4, which are the most potent D-peptides. On the other hand, both 4P1 and 4P2 inhibited roughly 55% of the enzymatic activity under such conditions. Our results indicate that the devised computational workflow successfully identified promising D-peptides displaying inhibitory potency against 3CL^pro^ in the micromolar concentration range.

### Identification of the Most Stable Binding Modes of 4P1, 4P2, 4P3, and 4P4 to 3CL^pro^ Combining Long MD Simulations, PCA, FEL and Clustering

Replicate 1 μs MD simulations were conducted for 3CL^pro^ in complex with the experimentally-tested D-tetrapeptides to assess the time stability of binding modes previously proposed from shorter MD simulations (Figure 4) and the possible occurrence of alternate bound conformations. First, we noticed large deviations in the RMSD values for all 3CL^pro^ backbone atoms concerning the starting structures along several 1 μs trajectories (Supplementary Figure S6). However, it became apparent through visual inspection that such deviations are caused by large motions of domain III relative to the ChT-like domains. This was corroborated after calculating the RMSD for the backbone atoms of ChT-like domains, as very stable time profiles were obtained in this case (Supplementary Figure S6). Moreover, RMSF values for all 3CL^pro^ plus D-peptide backbone atoms calculated after fitting the trajectories in respect of the backbone atoms of ChT-like domains show the large relative fluctuations of domain III (Supplementary Figure S6), which are likely to arise from the fact that 3CL^pro^ was simulated in the monomeric state to reduce the computational demand. The relatively loose interactions between domain III and the ChT-like domains suggest that the latter might suffice to simulate complexes with active site ligands, thus increasing the MD simulations performance. On the other hand, the RMSF profiles sharply drop for residues beyond 306, which belong to the D-tetrapeptides (see region 3 in Supplementary Figure S6). Along with the intermolecular H-bond time profiles (Supplementary Figure S7), this result indicates that the D-tetrapeptides keep forming favorable interactions with 3CL^pro^ active site residues during all the simulation time.

**FIGURE 6:**
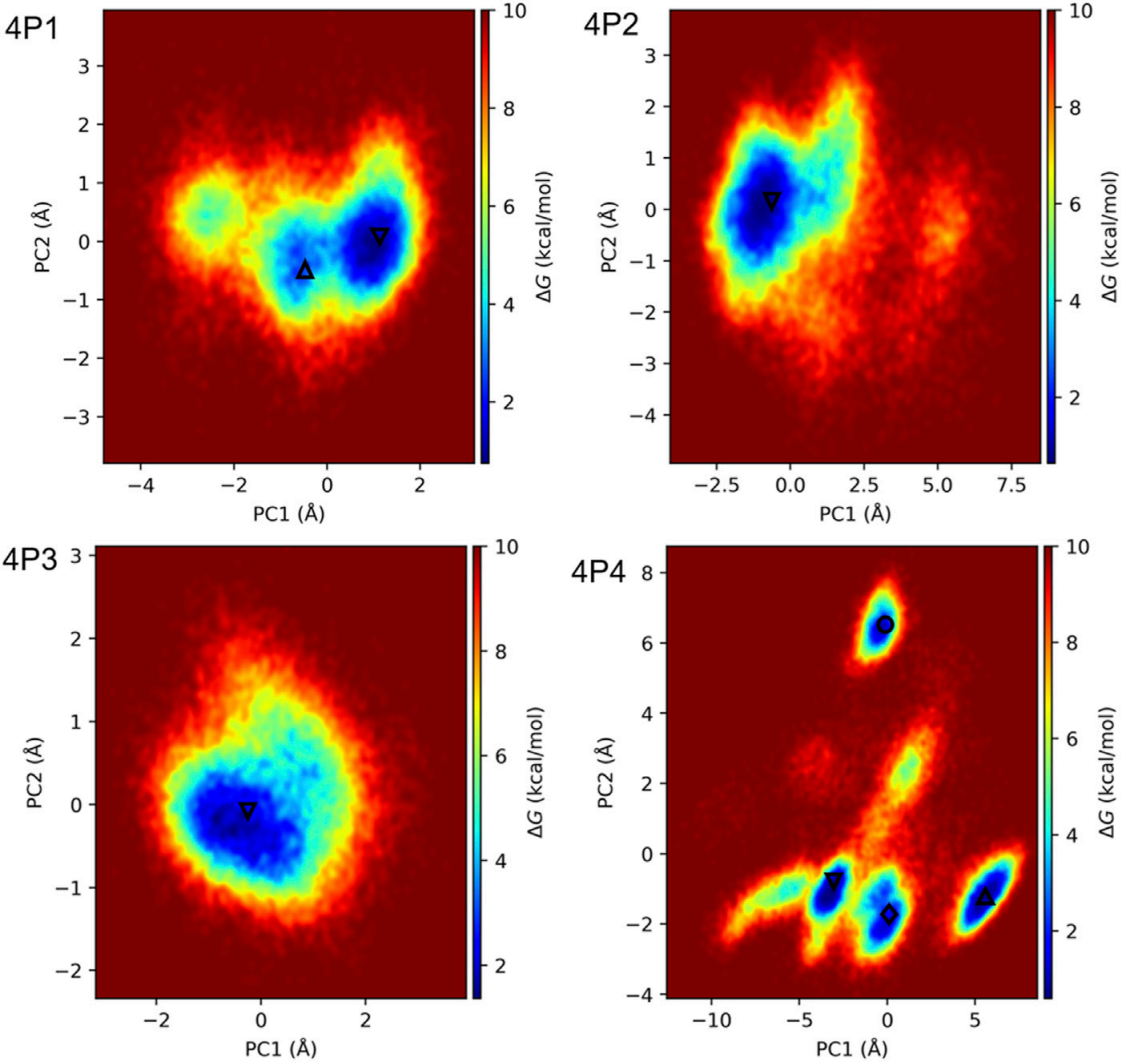
FEL heatmaps for 3CL^pro^ in complex with 4P1, 4P2, 4P3, and 4P4. FELs were obtained by projecting the concatenated replicate 1 μs trajectories of the complexes onto PC1 and PC2, associated with the motions of the D-peptide Cα atoms. All trajectories were fitted to the respective initial structures with respect to the backbone atoms of 3CL^pro^ in the ChT-like domains. The local energy minima observed in the heatmaps are indicated with the symbols Δ, ∇, ◊, and ○, which rank the minima according to their relative sizes in decreasing order (Δ > ∇ > ◊ > ○). PC1 and PC2 were chosen to generate FELS because they account for more than 68% of the motions of the D-peptide Cα atoms in all cases (Supplementary Figure S10).

The RMSD time profiles for 4P1 backbone atoms calculated after fitting the trajectories with respect to the backbone atoms of ChT-like domains indicate that the D-peptide remains bound in conformations similar to that of the starting structure throughout the microsecond-long MD simulations (Supplementary Figure S8). Nonetheless, we observed several transitions between slightly different conformations in both backward and forward directions during the replicate MD simulations of this complex. The previous result was confirmed by the FEL obtained by projecting the concatenated 1 μs trajectories onto PC1 and PC2 (Figure 6). Indeed, two main local minima or basins (termed 4P1-0 and 4P1-1, the former being more populated) are observed in the FEL heatmap (Figure 6). The 3CL^pro^/4P1 central structures corresponding to the main basins display slight differences in the conformation adopted by 4P1 ASN side-chain and ACE-GLY in the S1 and S2′ subsites, respectively (Supplementary Figure S9). Interestingly, the 4P1-1 conformation suggests that ASN(OD1) and ASN(ND2) at P1 can form alternate and less prevalent H-bonds with C145(N) and S144(OG), respectively (Supplementary Figure S9).

The long MD simulations for 3CL^pro^/4P2 and 3CL^pro^/4P3 complexes show stable peptide RMSD patterns (Supplementary Figure S8). FEL heatmaps in Figure 6 also indicate that 4P2 and 4P3 sampled conformations around a single energy basin during most of the simulation time. The central structures of both complexes corresponding to their respective minima (4P2-0 and 4P3-0) (Supplementary Figure S9) are very similar to those previously calculated from the 110 ns MD simulations (Figure 4). However, we found minor differences in the accommodation of 4P2-0 HIE side-chain at P1, which forms H-bonds with S144(O) instead of the H-bond with H163(NE2), more prevalent in the shorter MD simulation (Figure 4 and Supplementary Figure S9). Further calculations indicated that both H-bonds occur during the 2 μs concatenated MD simulations with 67 and 20% occupancies, respectively. Moreover, the 4P2-0 GLY residue at S2′ interacts preferentially with T26(OG1) rather than with G143(N) and C145(N) (Figure 4 and Supplementary Figure S9). On the other hand, no appreciable differences between 4P3-0 and the central structure of 4P3 corresponding to the shorter MD simulation are observed (Figure 4 and Supplementary Figure S9).

Contrary to what was observed for the previously-analyzed complexes, significant variations in the peptide RMSD patterns occur along the two replicate 1 μs MD simulations of the 3CL^pro^/4P4 complex (Supplementary Figure S8). Because of such instabilities, a third 1 μs MD simulation was run for this complex, which also shows wide peptide RMSD variations (Supplementary Figure S8). In agreement with the depicted RMSD patterns, the FEL for 3CL^pro^/4P4 indicates the existence of four main energy minima, termed 4P4-0 to 4P4-3, involving relatively large motions along PC1 and PC2 (Figure 6). The central structures corresponding to those minima also display appreciable divergence, especially 4P4-1 and 4P4-3 with respect to 4P4-0 and 4P4-2 (Supplementary Figure S9).

In principle, the stability of the sampled minima can be estimated from their relative abundances, i.e., the fraction of trajectory frames belonging to each minimum. However, under-sampling can still occur even in microsecond-long simulations, especially if transitions between different states do not occur several times in both directions during the simulation time, as in the present case (Supplementary Figure S8). This issue precludes the accurate calculation of conformational population sizes at equilibrium. Therefore, we decided to calculate the binding free energies (Δ*G*_*bind*_) for the four main conformations of the 3CL^pro^/4P4 complex sampled during the long MD simulations (see Supplementary Text S1 and references cited therein). Interestingly, the results show that conformations 4P4-0 and 4P4-2 have similar Δ*G*_*bind*_ values, considering the uncertainties of the calculated mean values (−5.2 ± 0.4 and −5.8 ± 0.4 kcal/mol, respectively, Supplementary Table S2) and the errors of ∼1 kcal/mol associated with the employed technique (Aldeghi et al., 2016). Of note, inhibition constants (*K*_*i*_) ranging from 50 to 100 μM are expected from the previous Δ*G*_*bind*_ values, in agreement with the micromolar inhibition potency displayed by 4P4 (Figure 5). The other two conformations, i.e., 4P1-1 and 4P4-3, are significantly less stable (−0.8 ± 0.4 and −1.9 ± 0.3 kcal/mol, respectively, Supplementary Table S2). Overall, the free energy calculations show that the relative stabilities of the different sampled conformations do not match the results expected from the relative sizes of the four main energy minima observed in the 3CL^pro^/4P4 FEL.

The two lowest-energy and nearly-isoenergetic conformations of 4P4 (4P-0 and 4P4-2, Supplementary Figure S9) differ mainly in the accommodation of HIE1 and ACE in S′ side of the active site. In fact, 4P4-0 HIE1 forms an H-bond with C44(O), whereas 4P4-2 HIE1 forms an H-bond with H41(O) (Supplementary Figure S9). The remaining residues in both conformations occupy the same subsites, although some small differences in their positions are observed (Supplementary Figure S9). On the other hand, the 3CL^pro^/4P4 central structure determined from the 110 ns MD simulation (Figure 4) adopts roughly the same conformation of 4P4-0 HIE1 at S1′, whereas the accommodation of the remaining residues in the former structure resembles to a larger extent that of 4P4-2 (Figure 4 and Supplementary Figure S9).

## Discussion

This work reports the *in silico* identification and *in vitro* validation of promising D-peptide inhibitors of SARS-CoV-2 3CL^pro^. An in-house D-peptide library was built from scratch to search for potential 3CL^pro^ inhibitors through a computational workflow comprising SBVS with AutoDock Vina and several rescoring steps (Figure 1). This workflow was already employed elsewhere to predict nonpeptidic allosteric inhibitors against the malarial protease falcipain-2 (Hernandez Gonzalez et al., 2021) and was adapted here for D-peptide SBVS. To our knowledge, our study is the first to employ a docking-based approach to screen D-peptide libraries against protein targets.

Protein/peptide docking remains a challenging task due to the high flexibility of peptides, which undermines the prediction of accurate bound conformations (Rentzsch and Renard, 2015; Ciemny et al., 2018; Hashemi et al., 2021). To address this issue, existing protein/peptide docking methodologies have relied on different strategies. Peptide poses can be generated on the fly, and the most favorable ones can be selected according to their binding energy scores (Morris et al., 1998; Ewing et al., 2001; Staneva and Wallin, 2009; Hashemi et al., 2021). However, this approach, coined as *de novo* docking, is not suitable for larger peptides, as exhaustive conformational sampling becomes prohibitive due to the presence of many freely-rotatable covalent bonds (Yan et al., 2017; Ansar and Vetrivel, 2019). This hurdle can be potentially overcome by conducting ensemble docking, which involves the generation of peptide conformations that can be subsequently docked into the protein binding site through rigid docking (Yan et al., 2017; Ciemny et al., 2018; Zhou et al., 2018; Ansar and Vetrivel, 2019). Moreover, MD simulation-based refinement steps can improve the accuracy of docked peptide poses (Trellet et al., 2013; Schindler et al., 2015; Yan et al., 2016; Ciemny et al., 2018; Hashemi et al., 2021).

Despite the availability of multiple peptide/protein docking tools (Ciemny et al., 2018; Hashemi et al., 2021), their efficient integration into SBVS campaigns is not straightforward (Ansar and Vetrivel, 2019). Recently, Ansar and Vetrivel presented PepVis, a pipeline for peptide SBVS following an ensemble docking approach (Ansar and Vetrivel, 2019). Their pipeline involves the generation of multiple peptide conformations that are rigidly docked into the targets and subsequently rescored and refined. As in PepVis, the D-peptide library screened in our work was created from the sequence and the starting structures were solvated and energy-minimized. However, to keep things simpler, we intentionally built very small D-peptide (three and four residues) libraries, thus avoiding issues like predicting the peptide secondary structure (Ansar and Vetrivel, 2019). This was possible in our case, as we concluded by visually inspecting several crystal structures of 3CL^pro^ in complex with peptidomimetic inhibitors that D-tripeptides and especially D-tetrapeptides are large enough to occupy the main pockets of the enzyme’s active site. Given the small size of the screened D-peptides, the ensemble approach was deemed unnecessary as it would have required additional computational steps. Instead, bound conformations during SBVS were generated and scored by the docking algorithm, as customary for small molecules. Subsequent rescoring steps in our workflow, such as generating multiple poses with AutoDock Vina using increased exhaustiveness of the search and different random seeds and MD simulations, aimed to enhance the conformational sampling of the analyzed D-peptides.

To prepare and screen libraries of larger D-peptides starting from the sequence, additional steps related to the peptide structure prediction and conformational search will be required. Existing methods and pipelines devised for protein/peptide docking (Yan et al., 2017; Ciemny et al., 2018; Ansar and Vetrivel, 2019; Hashemi et al., 2021) can be readily used if mirror images of the L-peptide structures (Garton et al., 2018) are created prior to the search for bound poses. However, the most straightforward strategy, inspired by mirror-image phage display experiments to identify D-peptide ligands (Schumacher et al., 1996), is to invert the configuration of the target protein Cα atoms and use an L-peptide library for SBVS.

The D-peptides proposed here as 3CL^pro^ inhibitors are mostly made up of aromatic residues plus HIS. Initially, we were intrigued by the fact that LEU was not found in any position of the selected D-peptides, although both SARS-CoV and SARS-CoV-2 3CL^pro^’^s^ prefer this residue at P2 (Chuck et al., 2010; Rut et al., 2021). However, at least in multiple heterochiral peptides reported by Rut et al., the preference for LEU at P2 is restricted to the L-enantiomer (Rut et al., 2021), which might explain why LEU is absent from the identified D-peptides. Furthermore, the predicted variable accommodation of D-peptide backbones along the 3CL^pro^ active site, divergent from the canonical conformation of the L-peptides (Figure 3), implies that specificity profiles obtained for L-peptide substrates cannot be straightforwardly extrapolated to small D-peptides. Finally, it is worth noting that the S2 subsite of 3CL^pro^ can accommodate bulky aromatic moieties, e.g., 3-fluoro-L-PHE (PDB: 6M0K) (Dai et al., 2020), 4-nitro-L-phenylalanine (PHE(4-NO_2_)) and 2,3-dihydro-L-tryptophane (Dht) (Rut et al., 2021), thus demonstrating that there is room in this subsite for large residues like those observed in the predicted D-peptides.

Apart from the previous factors, the absence of aliphatic residues in the identified D-peptides in favor of aromatic residues may arise from a persistent bias in our workflow toward larger ligands. In fact, it is known that Autodock Vina and other docking algorithms tend to overestimate the affinity of large ligands (Chang et al., 2010). Nonetheless, as mentioned before, the impact of such bias on the final results was reduced by the subsequent rescoring steps combining MM-GBSA free energy calculations and MD simulations. Interestingly, in a previous work, two L-peptides, HHYWH and HYWWT, identified as potential 3CL^pro^ inhibitors using AutoDock Vina (Porto, 2021), showed a high content of HIS, TYR, and TRP, in resemblance to our results. This coincidence seems to reinforce the occurrence of a bias toward the former residues in the docking algorithm.

Despite the inaccuracies of the employed computational techniques pointed out earlier, the inhibition assays carried out for the four top-ranked D-peptides (4P1, 4P2, 4P3, and 4P4) validated our predictions. The tested D-tetrapeptides displayed significant inhibition of 3CL^pro^ activity at 20 μM, causing 55–85% loss of activity in all cases. Moreover, the FELs obtained from microsecond-long MD simulations conducted for 3CL^pro^ bound to 4P1, 4P2, and 4P3 showed the stability of such complexes during the simulation time and that they sampled conformations around one or two similar main energy minima. Conversely, 4P4 sampled several well-separated energy minima and relatively-large peptide RMSD variations along the replicate 1 μs MD trajectories. However, further energetic analyses indicated that this D-peptide coexists as two nearly-isoenergetic conformations with binding free energies consistent with its experimental inhibitory potency. In general, the main conformations of the tested D-peptides obtained from the long MD simulations were similar to those of the 110 ns MD simulations that were conducted as part of the presented *in silico* workflow. Therefore, we believe that short MD simulations are sufficient to identify promising ligands. On the other hand, longer MD simulations can be conducted after experimental validation to predict more accurate complex conformations that can be used as starting points for structure-based optimization of the hits.

The predicted structures of 3CL^pro^ in complex with the selected D-peptides indicate the occurrence of significant intermolecular H-bonds present in the available crystal structures of this protease and its close homologue SARS-CoV 3CL^pro^. In this sense, we observed the formation of H-bonds between the amide oxygen of ASN/GLN of the D-peptides and H163(NE2), which explains the strong preference for GLN at P1 (Singh et al., 2020), in several predicted complexes, including that of the tested D-peptide 4P1. Other D-peptides, such as 3P1 and 4P2, were found to accommodate THR and HIE at the S1 subsite and to either mimic the H-bonds formed between GLN in L-peptides and H163(NE2) or to form alternate stabilizing interactions within the subsite. Of note, D-amino acids with side-chains smaller than GLN could reach the bottom of the S1 subsite because the backbones of the analyzed D-peptides can lie closer to the pocket entrance than the L-peptide backbones. Overall, the previous results show that our workflow was able to capture the interactions underlying the fine-tuned specificity of the S1 subsite of 3CL^pro^ (Rut et al., 2021).

D-peptides are considered attractive therapeutic agents (Liu et al., 2016). However, this type of molecule has not been explored as potential ligands of 3CL^pro^ active site until now. Therefore, the tested D-tetrapeptides 4P1, 4P2, 4P3, and 4P4 expand the chemical repertoire of known 3CL^pro^ inhibitors that can help combat Covid-19. Beyond this concrete example, the computational workflow presented here can contribute to the fast discovery of small D-peptide ligands targeting different 3CL^pro^ variants that can arise under viral adaptation to drug pressure (Padhi and Tripathi, 2021), as well as other proteins of interest.

## Data Availability

The original contributions presented in the study are included in the article/Supplementary Material, further inquiries can be directed to the corresponding author.
