# Supplementary material for: A Computer-Aided Approach for the Discovery of D-Peptides as Inhibitors of SARS-CoV-2 Main Protease
Source: Front Mol Biosci. 2022 Jan 24;8:816166. doi: 10.3389/fmolb.2021.816166 (PMC8852625; doi:10.3389/fmolb.2021.816166)
Supplement: Supplementary file 1 [file DataSheet1.pdf]

Supporting Information

A computer-aided approach for the discovery of D-peptides as inhibitors of SARS-CoV-2 main protease

Jorge E. Hernández González[1,2], Raphael J. Eberle[3,4], Dieter Willbold[3,4,5], Mônika A. Coronado[3,*]

[1] Multiuser Center for Biomolecular Innovation, IBILCE, Universidade Estadual Paulista (UNESP), São Jose do Rio Preto-SP, Brazil

[2] Laboratory for Molecular Modeling and Dynamics, Instituto de Biofísica Carlos Chagas Filho, Universidade Federal do Rio de Janeiro, Ave. Carlos Chagas Filho, 373, CCS-Bloco D sala 30, Cidade Universitária Ilha de Fundão, Rio de Janeiro, CEP 21941-902, Brazil.

[3] Institute of Biological Information Processing (IBI-7: Structural Biochemistry), Forschungszentrum Jülich, Jülich, Germany.

[4] Institut für Physikalische Biologie, Heinrich-Heine-Universität Düsseldorf, Universitätsstraße, Düsseldorf, Germany.

[5] JuStruct: Jülich Centre for Structural Biology, Forschungszentrum Jülich, Jülich, Germany.

*** Correspondence:**

Corresponding Author m.coronado@fz-juelich.de

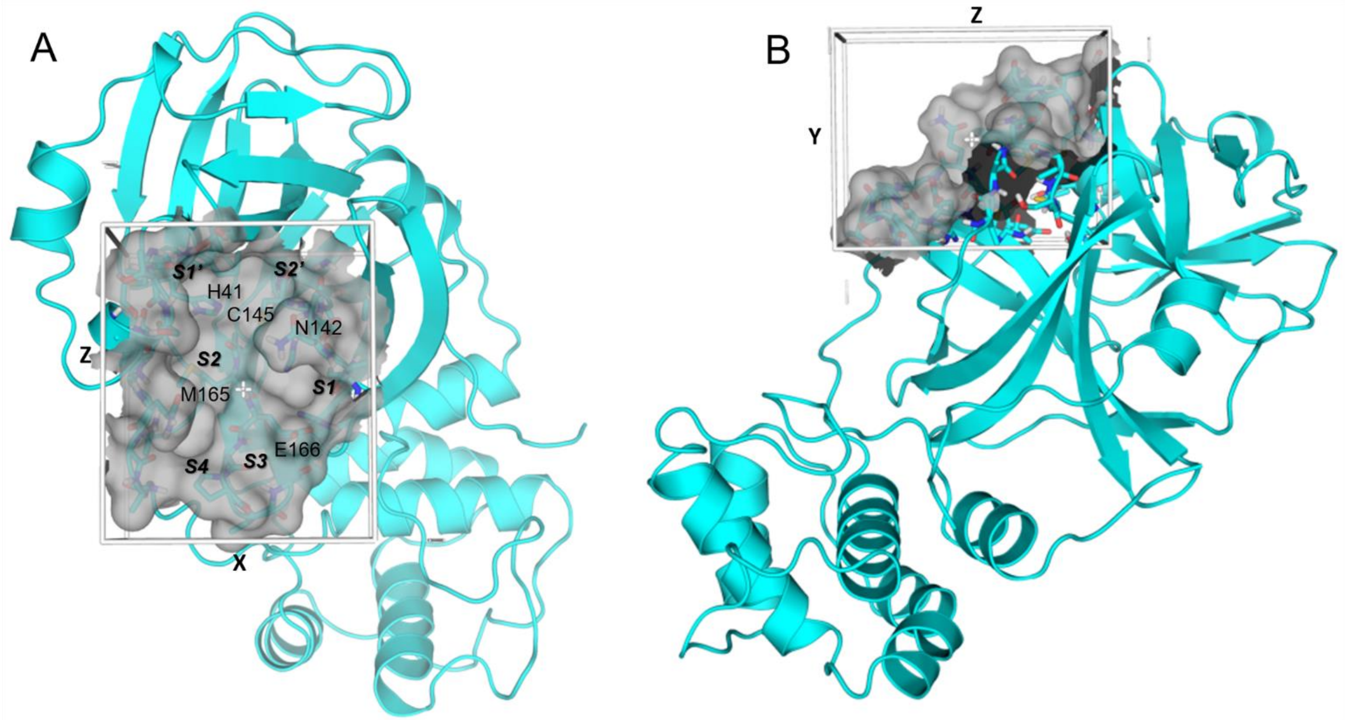

Supplementary Figure S1. Cuboid docking box built with Autodock/Vina plugin of Pymol (DeLano, 2002; Seeliger and de Groot, 2010) and used for SBVS seen from **A)** a top view and **B)** a side view. The box dimensions in X, Y and Z axes are 19.5, 18.0 and 22.0 Å, respectively. The 3CL^{pro} active site inside the docking box is shown in surface representation. Key residues of the active site and subsites are labeled, the latter being highlighted in bold. The crystal structure of 3CL^{pro} PDB 6Y2E was employed for SBVS.

Supplementary Table S1. Details of the MD simulations performed for $3CL^{pro}$ in complex with the D-peptides proposed as inhibitors[a]

System	Number of TIP3P waters	Solute net charge	Number of Counter-ions
$3CL^{pro}$/3P1	16,550	4-	4 Na^+
$3CL^{pro}$/3P2	16,602	4-	4 Na^+
$3CL^{pro}$/3P3	16,553	4-	4 Na^+
$3CL^{pro}$/3P4	16,522	4-	4 Na^+
$3CL^{pro}$/3P5	16,562	4-	4 Na^+
$3CL^{pro}$/3P6	16,571	4-	4 Na^+
$3CL^{pro}$/4P1	16,564	4-	4 Na^+
$3CL^{pro}$/4P2	16,557	4-	4 Na^+
$3CL^{pro}$/4P3	16,561	4-	4 Na^+
$3CL^{pro}$/4P4	16,567	4-	4 Na^+
$3CL^{pro}$/4P5	16,555	4-	4 Na^+
$3CL^{pro}$/4P6	16,791	4-	4 Na^+
$3CL^{pro}$/4P7	16,694	4-	4 Na^+
$3CL^{pro}$/4P8	16,559	4-	4 Na^+
$3CL^{pro}$/4P9	16,541	4-	4 Na^+
$3CL^{pro}$/4P10	16,588	3-	3 Na^+

[a]For brevity's sake only the complexes identified after the completion of the workflow outlined in Figure 1 and shown in Table 1 are included here. D-peptides are termed according to the nomenclature used in Table 1.

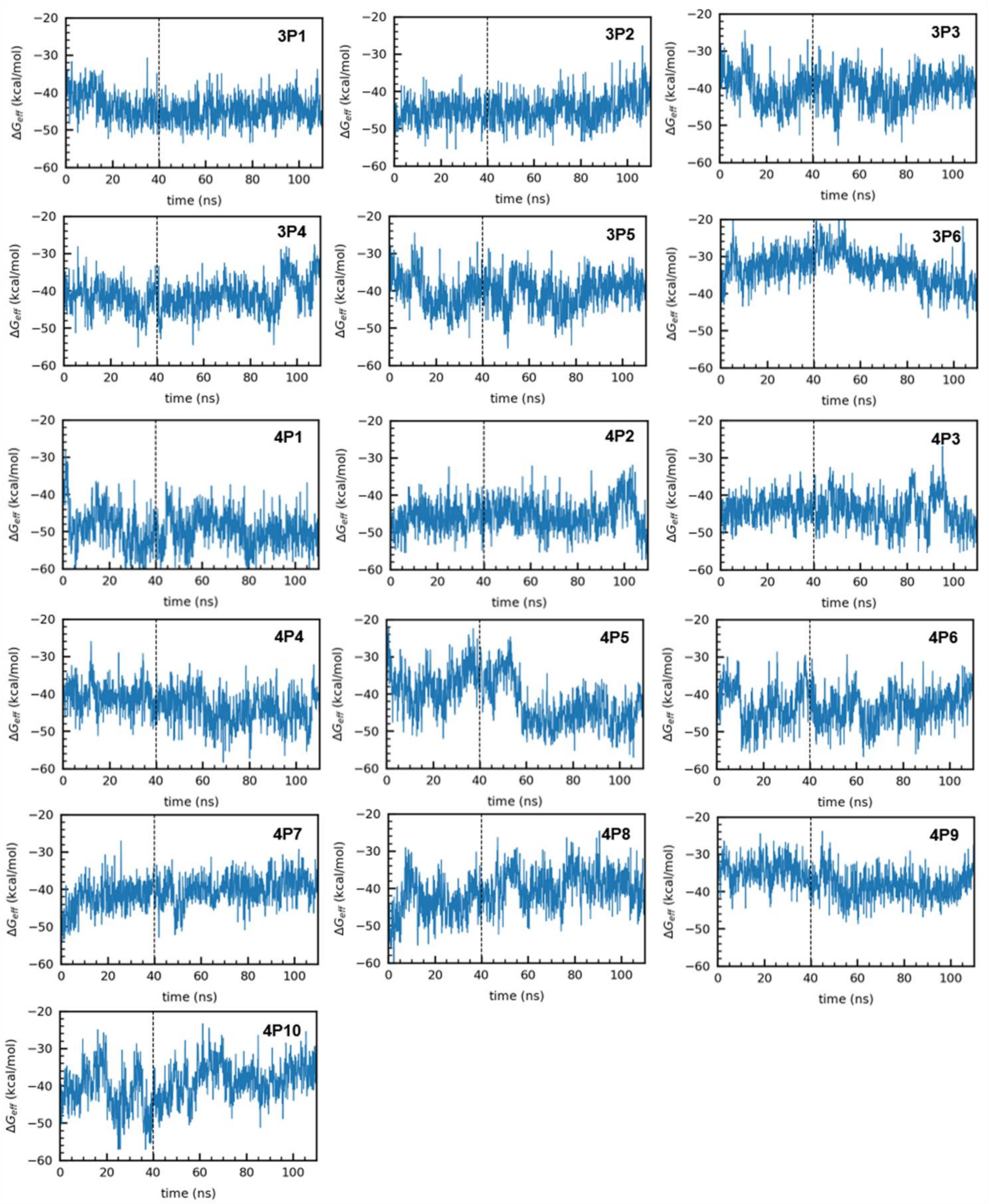

Supplementary Figure S2. ΔG_{eff} time profiles along the 110 ns trajectories of 3CL^{pro} in complex with the D-peptides selected as potential inhibitors. Values collected after 40 ns (see dashed lines) were used to calculate the reported ΔG_{eff} mean values for the complexes. D-peptides are labeled as in Table 1.

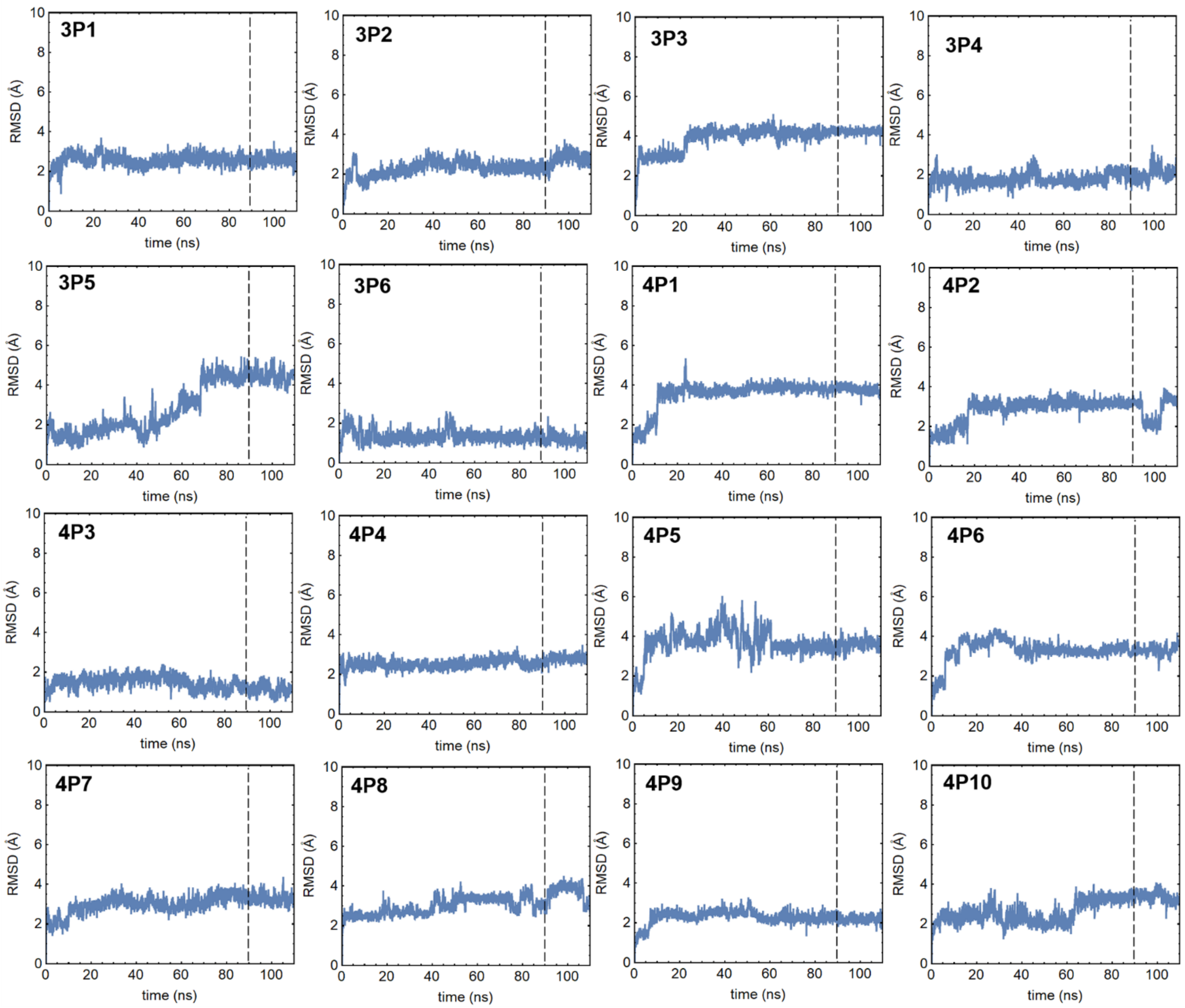

Supplementary Figure S3. Peptide RMSD time profiles along the 110 ns trajectories of $3CL^{pro}$ in complex with the D-peptides selected as potential inhibitors. RMSD values were calculated for the D-peptide heavy atoms after fitting all trajectory frames to the ChT-like domains backbone atoms of $3CL^{pro}$ in the corresponding starting structures (t=0). Values collected after 90 ns (see dashed lines) were used to calculate the reported peptide RMSD mean values for the complexes. D-peptides are labeled as in Table 1.

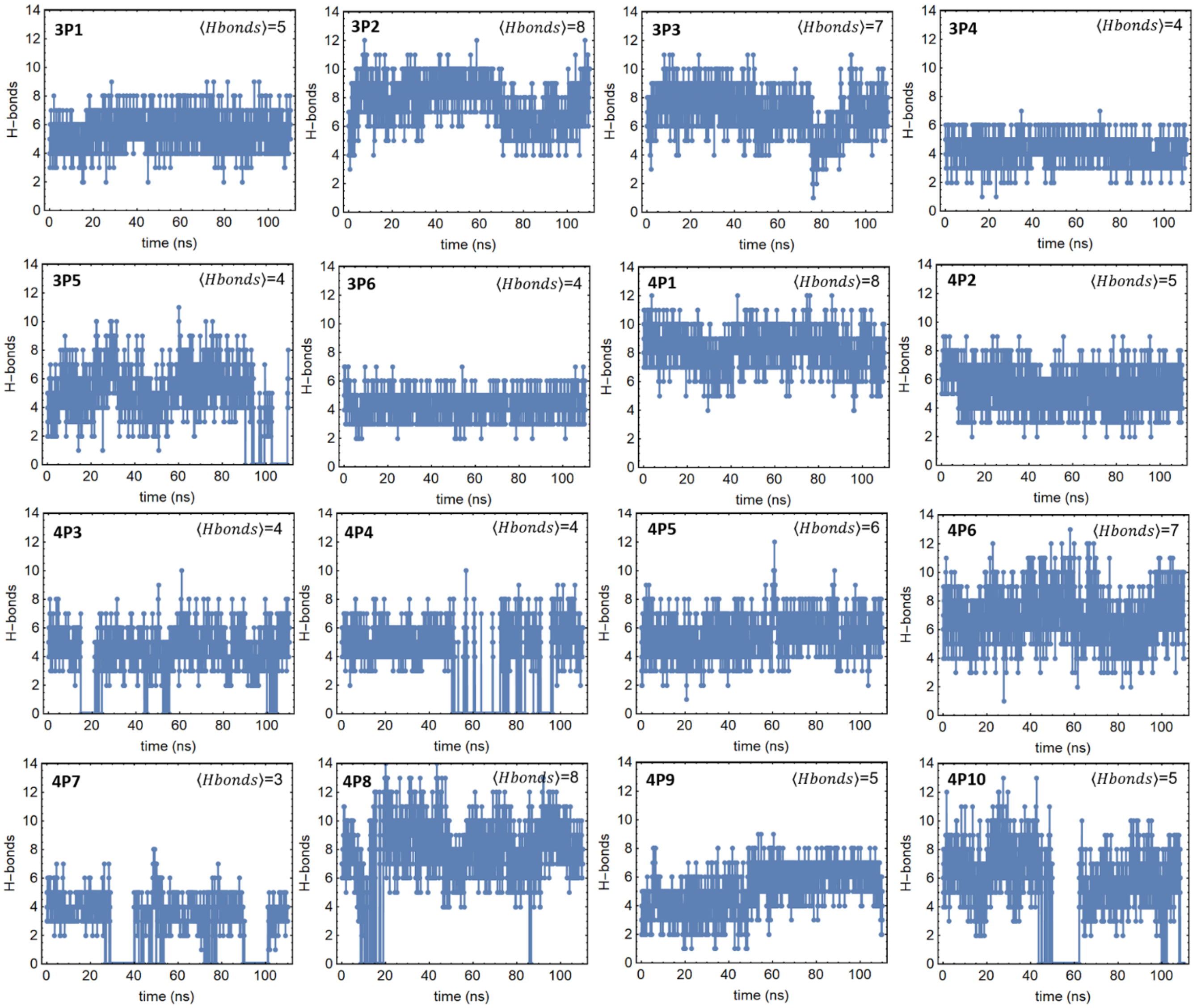

Supplementary Figure S4. Number of intermolecular H-bonds between the indicated D-peptides and $3CL^{pro}$ residues during the 110 ns MD simulations. The geometric criteria to determine the occurrence of an H-bond was a donor-acceptor distance $\leq$ 3.5 Å and a donor-H-acceptor angle $\geq$120°. $\langle Hbonds \rangle$ represents the average number of intermolecular H-bonds formed along each trajectory.

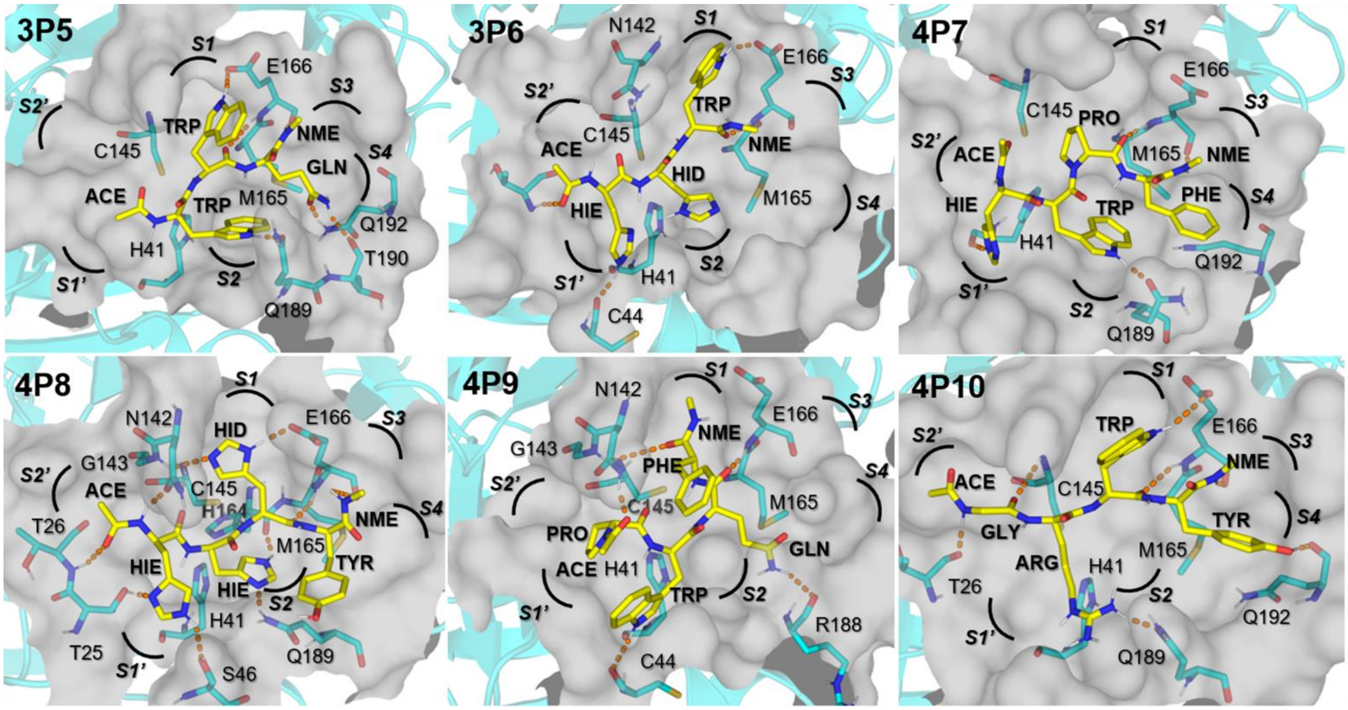

Supplementary Figure S5. Structural representation of 3CL^{pro} in complex with D-peptides proposed as potential inhibitors and with ΔG_{eff} values >-40 kcal/mol. All D-peptides are shown as yellow sticks and their residues are labeled in bold and in the three-letter code. 3CL^{pro} residues forming H-bonds with the peptides plus the catalytic residues H41 and C145 are labeled and represented as cyan sticks. The 3CL^{pro} active site cavity is depicted as a transparent gray surface. H-bonds between the D-peptides and the 3CL^{pro} residues with occupancies >25% during the respective 110 ns MD simulations are displayed as orange dashed lines. Subsites S4 to S2' are labeled in bold and italic.

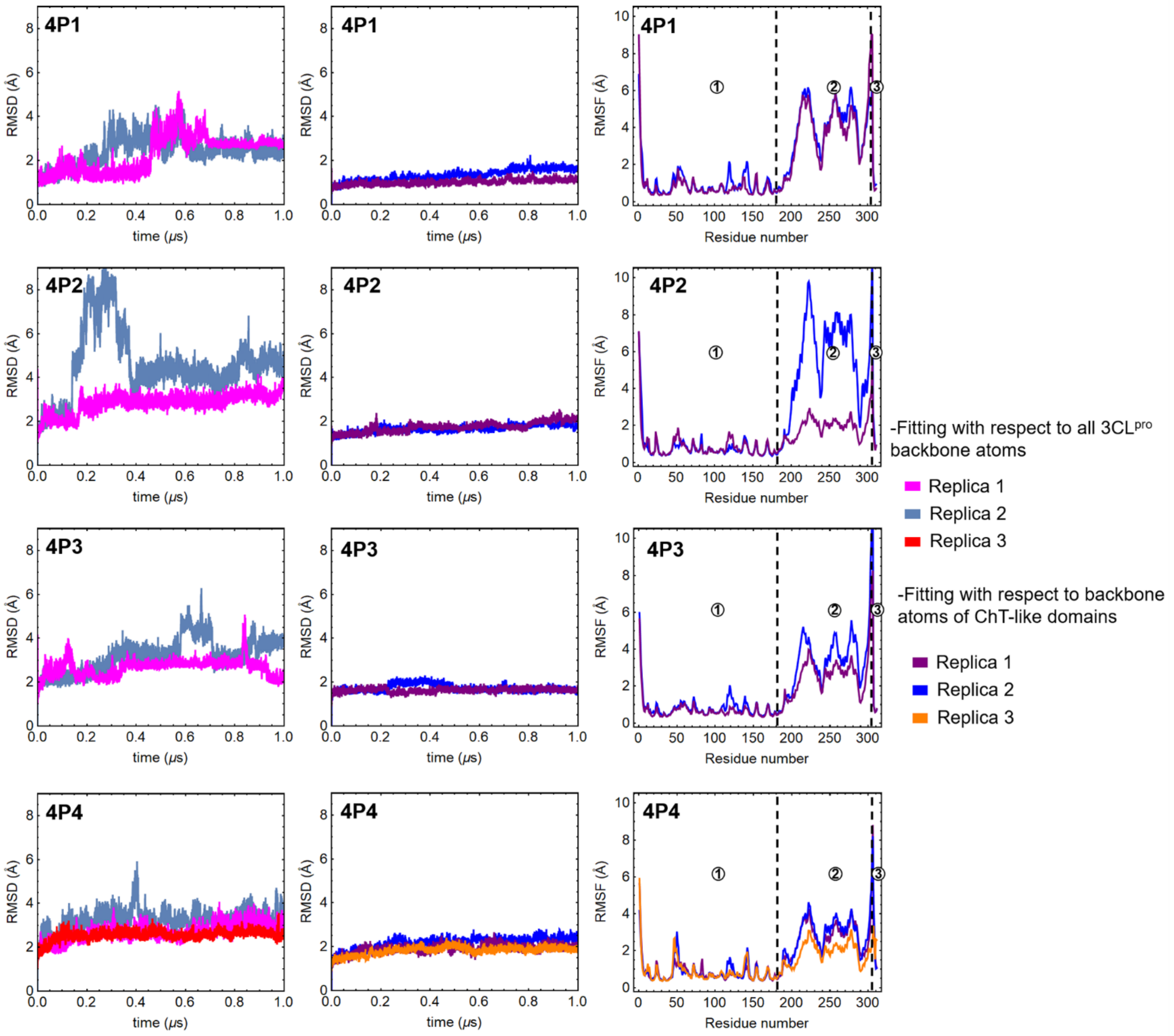

Supplementary Figure S6. Instantaneous RMSD values and RMSF profiles for the replicate 1 μs MD trajectories of 3CL^{pro} in complex with the experimentally-tested D-peptides. For every complex, from left to right, the RMSD time profiles with respect to all backbone atoms of 3CL^{pro}, the RMSD time profiles with respect to the backbone atoms of 3CL^{pro} ChT-like domains (residues 8 to 183), and the RMF profiles with respect to all backbone atoms in the 3CL^{pro}/D-peptide complexes are represented. The three regions labeled 1, 2 and 3 in the RMSF graphs correspond to the ChT-like domains, domain III and the D-tetrapeptides, respectively. Profiles corresponding to different replicate MD simulations are colored differently (see legend on the right). RMF values were calculated after fitting the trajectories with respect to the backbone atoms of the ChT-like domains. In all cases, the respective starting structures (t=0) were set as reference for fitting.

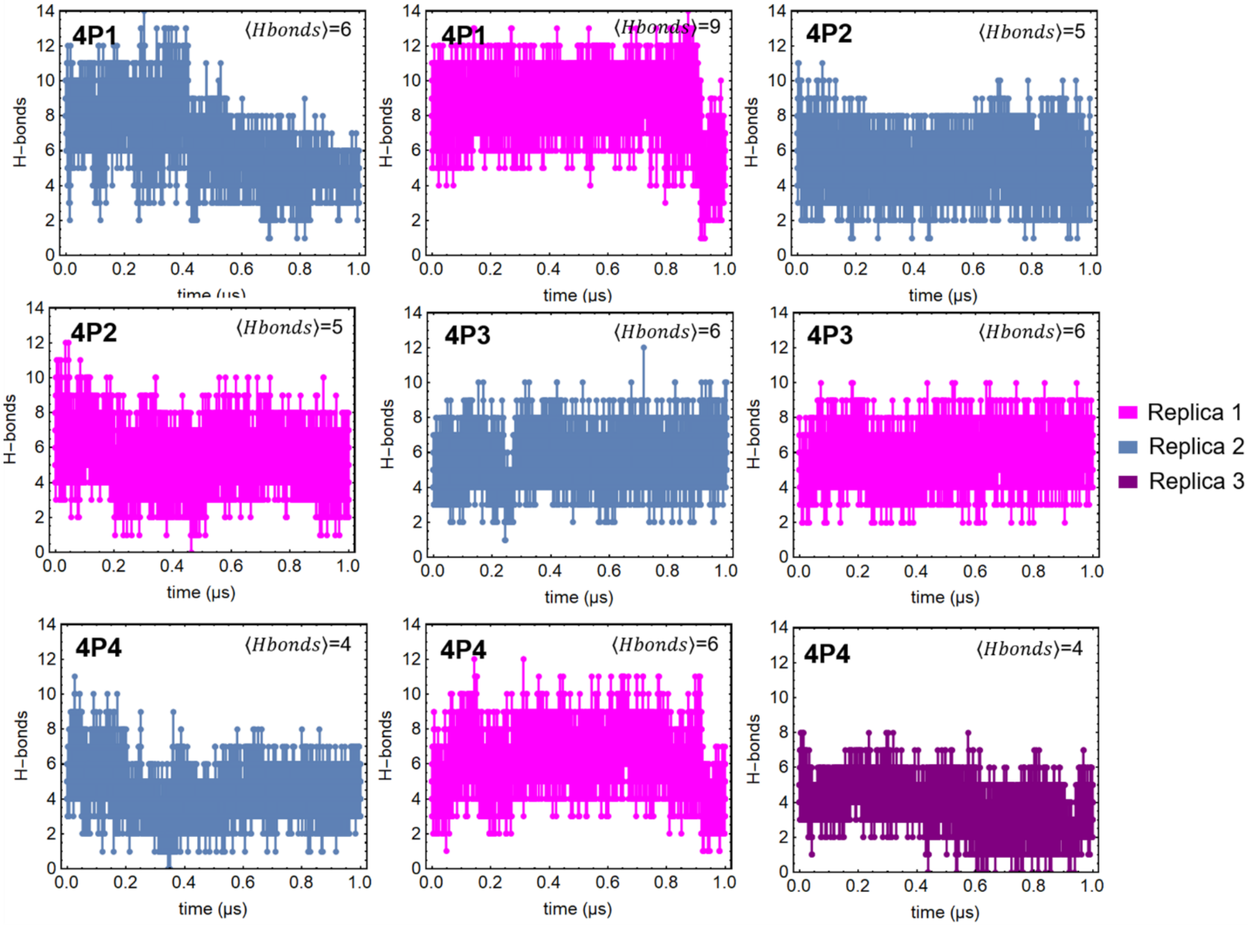

Supplementary Figure S7. Number of intermolecular H-bonds between the experimentally-tested D-peptides and 3CL^{pro} residues during replicate 1 μs MD simulations. The geometric criteria to determine the occurrence of an H-bond was a donor-acceptor distance $\leq$ 3.5 Å and a donor-H-acceptor angle $\geq$120°. $\langle Hbonds \rangle$ represents the average number of intermolecular H-bonds formed along each trajectory. Time profiles corresponding to different replicate MD simulations are colored differently (see legend on the right).

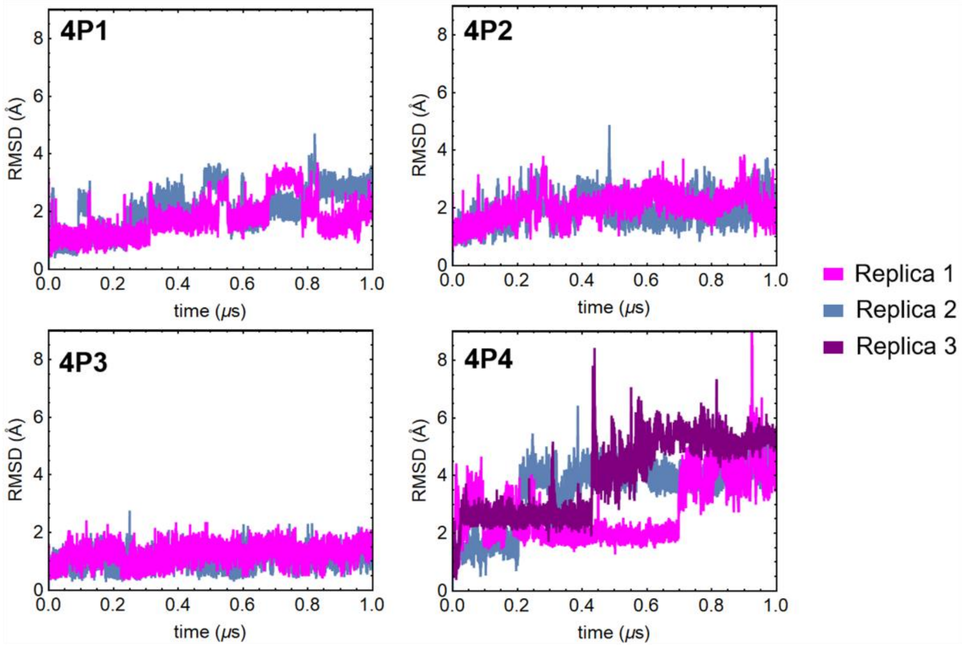

Supplementary Figure S8. Peptide RMSD values along the replicate 1 μs MD simulations of $3CL^{pro}$ in complex with the experimentally-tested D-tetrapeptides. RMSD values were calculated for the peptide heavy atoms after fitting all trajectory frames with respect to the backbone atoms of $3CL^{pro}$ residues 8 to 183 (chymotrypsin-like domains) in the corresponding starting structures. Time profiles corresponding to different replicate MD simulations are colored differently (see legend on the right).

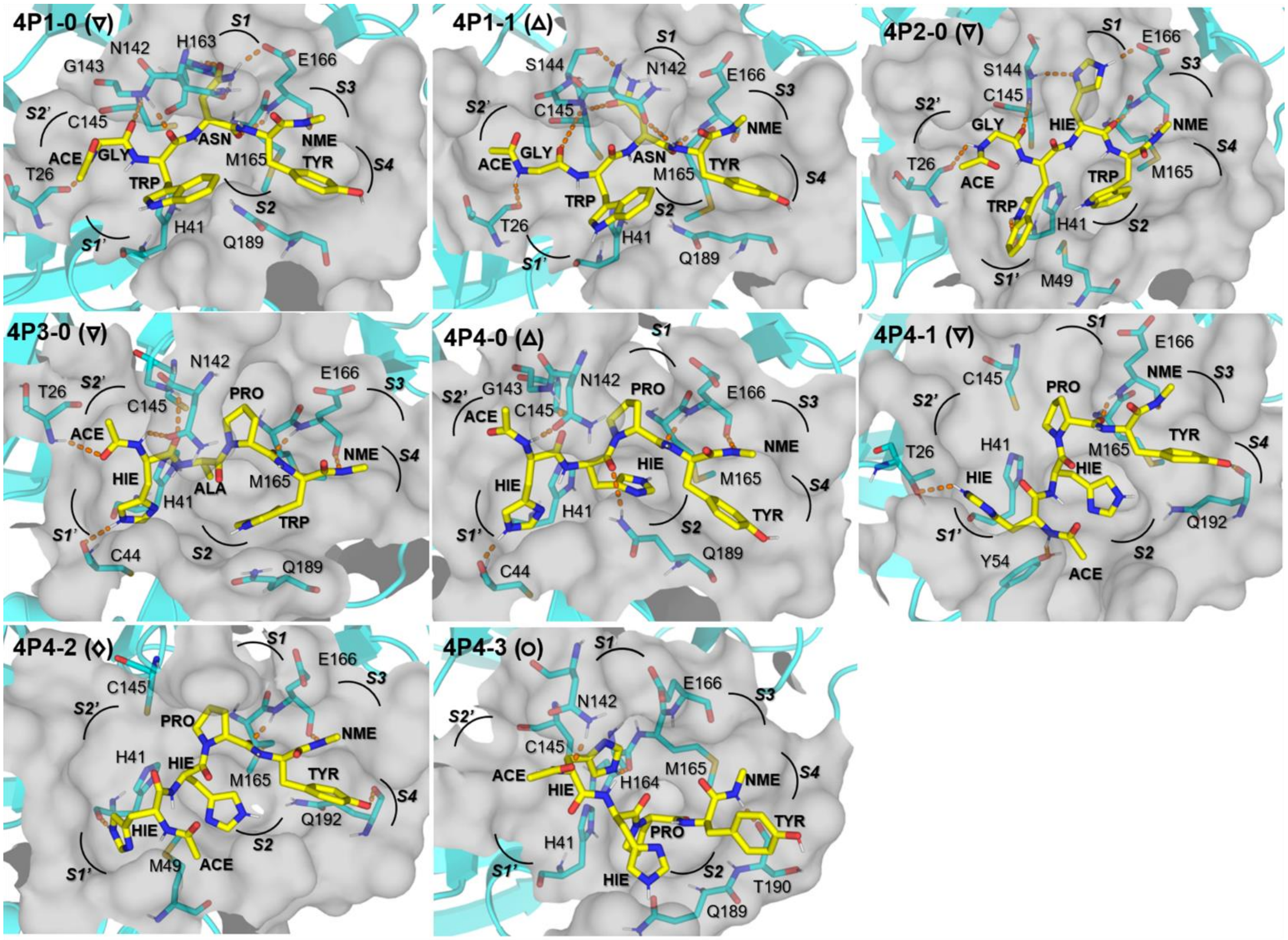

Supplementary Figure S9. Main central structures of 3CL^{pro} in complex with the experimentally-tested D-peptides obtained from the replicate 1 μs MD simulations. Symbols in parentheses indicate which minimum of the PC1 vs PC2 FELs (Figure 6) corresponds to each represented central structure. Different central structures of the same complex are labeled by adding a hyphen and a numerical identifier (0, 1, 2, 3) to the peptide abbreviation. The lower the numerical identifier the higher the size of the cluster to which the central structure belongs. All D-peptides are shown as yellow sticks and their residues are labeled in bold and in the three-letter code. 3CL^{pro} residues forming H-bonds with the peptides plus the catalytic residues H41 and C145 are labeled and represented as cyan sticks. The 3CL^{pro} active site cavity is depicted as a transparent gray surface. H-bonds between the D-peptides and the 3CL^{pro} residues are displayed as orange dashed lines. Subsites S4 to S2' are labeled in bold and italic.

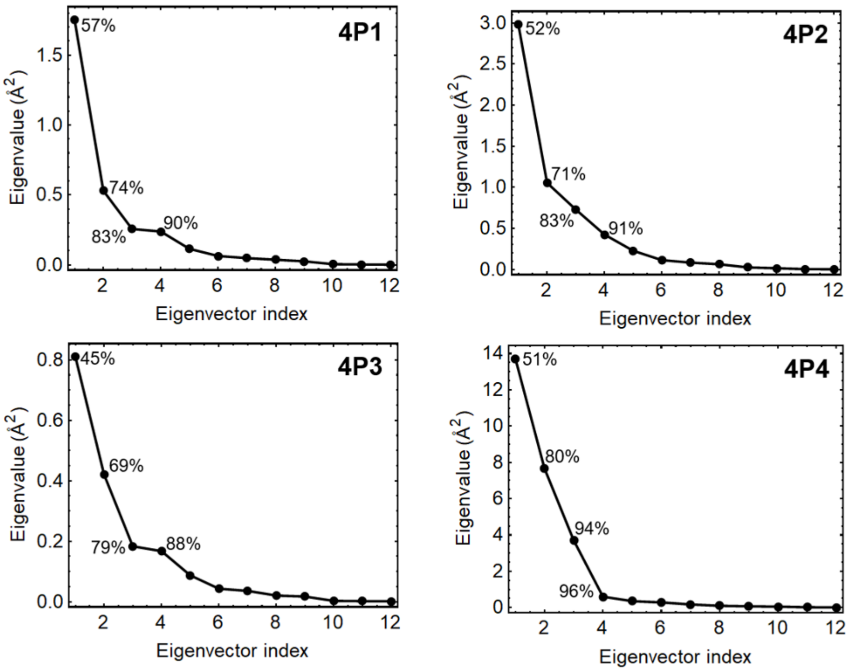

Supplementary Figure S10. Eigenvalues of the 12 eigenvectors corresponding to the concatenated 1 μs replicate MD simulations of 3CL^{pro} in complex with the experimentally-tested D-peptides. PCA was conducted for the D-peptide Cα atoms. The cumulative fluctuations of the first four eigenvectors are shown beside their corresponding points in each graph.

Supplementary Text S1. Thermodynamic integration free energy calculations

Thermodynamic integration (TI) binding free energy (ΔG^{o}_{bind}) calculations were conducted for the main conformations sampled by 4P4 bound to 3CL^{pro} (Supplementary Figure S9) to determine the lowest-energy binding mode. A protocol published by Aldeghi *et al.* is particularly suitable to identify lowest-energy binding modes of ligands to their receptors (Aldeghi et al., 2016). That protocol was previously modified by us to conduct the MD simulations with *pmemd.cuda* of Amber 20 (Case et al., 2020), and estimate ΔG^{o}_{bind} values using TI (Hernandez Gonzalez et al., 2021). Briefly, 4P4 was alchemically turned into non-interacting particles, i.e., dummy atoms, both free in solution and complexed with 3CL^{pro} in different starting conformations (Supplementary Figure S9) through a two-stage process. First, its atoms were progressively discharged until they all had zero partial charges. Then, 4P4 van der Waals parameters were turned off thus leading to a dummy ligand. The coupling parameter λ was varied from 0 to 1, with a Δλ=0.05 for both the discharging and van der Waals alchemical transformations. Each λ window was subjected EM, a 1 ns NVT heating and a 2 ns NPT equilibration to reach a temperature of 298.15 K and a pressure of 1 bar. Finally, 30 ns NPT production runs were conducted and the collected snapshots were used for energy calculations. To reduce the computational demand of these simulations, only the ChT-like domains of 3CL^{pro} bound to the different 4P4 conformations were chosen as starting structures. Moreover, hydrogen-mass repartitioning (Hopkins et al., 2015) was employed in order to increase the timestep from 2 to 4 fs during the NPT production runs. Pressure control was achieved by means of the Monte Carlo

barostat implemented in Amber 20 (Åqvist et al., 2004; Case et al., 2020). Other details of these MD simulations not mentioned here are identical to those described for conventional MD simulations in Materials and Methods. Free energies were finally calculated using the *alchemical-analysis.py* program, which performs autocorrelation analysis to determine the associated SEMs (Klimovich et al., 2015).

4P4 was decoupled from $3CL^{pro}$ active site in the presence of a set of restraints involving three atoms of the protein: H164(Cα) (a1), T25(Cα) (a2) and G146(Cα) (a3), and three 4P4 atoms: HIE1(Cα) (b1) PRO3(Cα) (b2) and TYR4(Cα) (b3) (Supplementary Figure S11). One distance restraint (a1-b1), k_r= 10 kcal·mol^{-1}·Å^{-2}, two angular restraints (a1-b1-b2 and a2-a1-b1), k_θ=20 kcal·rad^{-2}·mol^{-1}, and three dihedral restraints (a3-a2-a1-b1, a2-a1-b1-b2 and a1-b1-b2-b3), k_φ=20 kcal·rad^{-2}·mol^{-1} were set to keep the peptide bound to the active site while decoupled (Boresch et al., 2003). The energy contribution of setting those restraints to the decoupled D-peptide in the bound conformation (ΔG_{rst}^{dum}) was calculated through an analytical equation presented elsewhere (Boresch et al., 2003). Moreover, the free energy associated with the restraint removal when 4P4 is fully interacting with $3CL^{pro}$ (ΔG_{rst}^{int}) can be obtained through a single-step free energy perturbation (Doudou et al., 2009), as follows:

$$\Delta G_{rst}^{int} = RTln\,\langle exp\left(\frac{-k_r(r(t)-r_o)}{2RT}\right)\rangle_{k=0} + \sum_{i=1}^{2} RTln\,\langle exp\left(\frac{-k_{\theta,i}\left(\theta_i(t)-\theta_{o,i}\right)}{2RT}\right)\rangle_{k=0} + \sum_{i=1}^{3} RTln\,\langle exp\left(\frac{-k_{\varphi,i}\left(\varphi_i(t)-\varphi_{o,i}\right)}{2RT}\right)\rangle_{k=0} \quad \text{(S1)}$$

where k_r, $k_{\theta,i}$ and $k_{\varphi,i}$ are the harmonic distance, angular and dihedral restraint constants, respectively, which were all set to the values shown hereinbefore. $r(t)$, $\theta(t)$ and $\varphi(t)$ stand for the instantaneous values of the distances, angles and dihedrals between the selected atoms during the MD simulations at each λ value. The $\langle\ldots\rangle_{k=0}$ brackets mean that ensemble averages were calculated from unrestrained MD simulations. Finally, the subscripts i indicate that iteration over all angular and dihedral restraints is performed, and the subscripts o indicate the equilibrium restraint values, which were set according to the starting $3CL^{pro}$/4P4 conformation (Supplementary Figure S9).

Four unrestrained 30 ns MD simulations of each $3CL^{pro}$/4P4 conformation were performed to calculate ΔG_{rst}^{int} through Eq. S1. The results of the four replicates corresponding to each starting conformation were averaged and SEMs were reported.

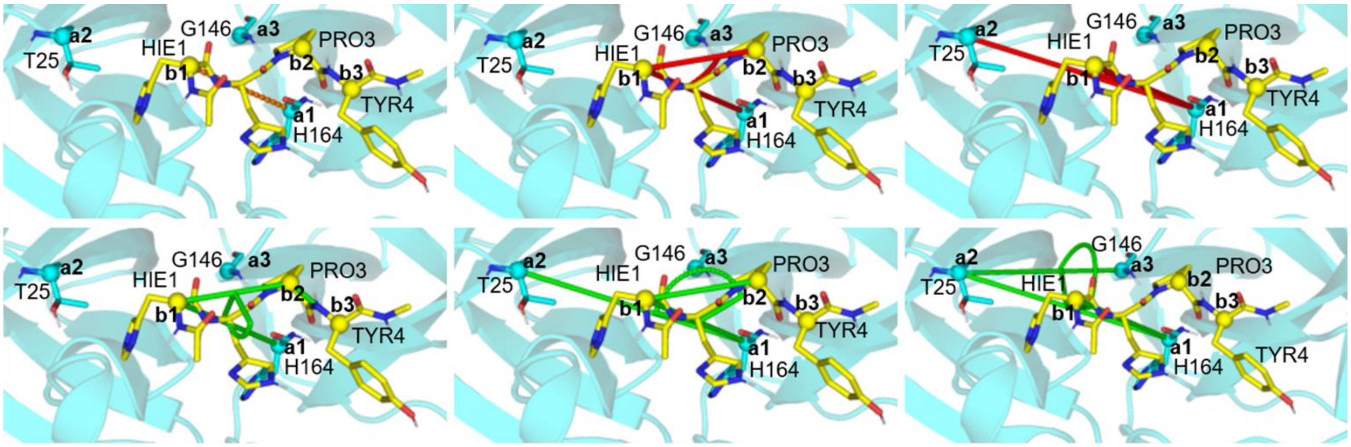

Supplementary Figure S11. Harmonic restraints applied to attach 4P4 to $3CL^{pro}$ active site during TI free energy calculations. Cα atoms chosen to apply the restraints are shown as spheres and labeled (a1 to a3 and b1 to b3). Distance, angular and dihedral restraints are represented as orange, red and green lines, respectively, linking the involved atoms. For brevity's sake, restraints are depicted for only one of the analyzed $3CL^{pro}$/4P4 conformations.

Supplementary Table S2. ΔG_{bind} values calculated for the different central structures of the $3CL^{pro}$/4P4 complex during the three replicate 1 μs MD simulations[a]

Central Structure	ΔG_{dech}^{rst} [b] (kcal/mol)	ΔG_{vdw}^{rst} [c] (kcal/mol)	ΔG_{rst}^{dum} [d] (kcal/mol)	ΔG_{rst}^{inte} [e] (kcal/mol)	ΔG_{bind}^{o} [f] (kcal/mol)
4P4-0 (Δ)	1.9±0.2	-13.0±0.2	7.71	-1.8±0.2	-5.2±0.4
4P4-1 (∇)	1.9±0.2	-8.7±0.3	7.44	-1.5±0.2	-0.8±0.4
4P4-2 (◊)	0.9±0.2	-11.9±0.2	7.53	-2.4±0.2	-5.8±0.4
4P4-3 (○)	4.5±0.2	-11.9±0.3	7.64	-2.1±0.1	-1.9±0.3

[a]Binding free energies were calculated using TI. The λ windows for the decharging and van der Waals steps were simulated for 30 ns, with a Δλ=0.05 and the first 10 ns were discarded.
[b]Free energy associated with the decharging steps of the different conformations of 4P4 bound to $3CL^{pro}$ in the presence of a set of harmonic restraints ($\Delta G_{dech}^{prot+rst}$) and free in solution ($\Delta G_{dech}^{free}$): $\Delta G_{dech}^{rst} = \Delta G_{dech}^{free} - \Delta G_{dech}^{prot+rst}$.
[c]Free energy associated with the disappearance of fully decharged 4P4 bound to $3CL^{pro}$ in the presence of a set of harmonic restraints ($\Delta G_{vdw}^{prot+rst}$) and free in solution ($\Delta G_{vdw}^{free}$): $\Delta G_{vdw}^{rst} = \Delta G_{vdw}^{free} - \Delta G_{vdw}^{prot+rst}$.
[d]Free energy variation upon the addition of a set of harmonic restraints attaching non-interacting (=dummy) 4P4 to the binding site, calculated through an analytical formula proposed by Boresch *et al.* (Boresch et al., 2003), that also contains the correction to the molar standard state (volume equal to 1661 $Å^3$).
[e]Free energy variation upon removal of the same set of restraints when the fully interacting D-peptide is bound to the protein, calculated using Eq. S1.
[f]Calculated standard binding free energies for each $3CL^{pro}$/4P4 central structure, obtained from the sum of the previous components.

References

Aldeghi, M., Heifetz, A., Bodkin, M.J., Knapp, S. and Biggin, P.C. (2016). Accurate calculation of the absolute free energy of binding for drug molecules. Chem Sci, 7, 207-218. doi:10.1039/c5sc02678d

Åqvist, J., Wennerström, P., Nervall, M., Bjelic, S. and Brandsdal, B.O. (2004). Molecular dynamics simulations of water and biomolecules with a Monte Carlo constant pressure algorithm. Chem Phys Lett, 384, 288-294. doi:10.1016/j.cplett.2003.12.039

Boresch, S., Tettinger, F., Leitgeb, M. and Karplus, M. (2003). Absolute Binding Free Energies: A Quantitative Approach for Their Calculation. J Phys Chem B, 107, 9535–9551. doi:10.1021/jp0217839

Case, D.A., Belfon, K., Ben-Shalom, I.Y., Brozell, S.R., Cerutti, D.S., Cheatham III, T.E., et al. (2020). Amber 2020. University of California, San Francisco

DeLano, W.L. (2002). PyMOL. San Carlos, CA, 700

Doudou, S., Burton, N.A. and Henchman, R.H. (2009). Standard Free Energy of Binding from a One-Dimensional Potential of Mean Force. J Chem Theory Comput, 5, 909-918. doi:10.1021/ct8002354

Hernandez Gonzalez, J.E., Salas-Sarduy, E., Hernandez Alvarez, L., Barreto Gomes, D.E., Pascutti, P.G., Oostenbrink, C., et al. (2021). In silico identification of noncompetitive inhibitors targeting an uncharacterized allosteric site of falcipain-2. J Comput Aided Mol Des, 35, 1067-1079. doi:10.1007/s10822-021-00420-7

Hopkins, C.W., Le Grand, S., Walker, R.C. and Roitberg, A.E. (2015). Long-Time-Step Molecular Dynamics through Hydrogen Mass Repartitioning. J Chem Theory Comput, 11, 1864-1874. doi:10.1021/ct5010406

Klimovich, P.V., Shirts, M.R. and Mobley, D.L. (2015). Guidelines for the analysis of free energy calculations. J Comput Aided Mol Des, 29, 397-411. doi:10.1007/s10822-015-9840-9

Seeliger, D. and de Groot, B.L. (2010). Ligand docking and binding site analysis with PyMOL and Autodock/Vina. J Comput Aided Mol Des, 24, 417-422. doi:10.1007/s10822-010-9352-6
